# Aggrandizement of fermented cucumber through the action of autochthonous probiotic cum starter strains of *Lactiplantibacillus plantarum* and *Pediococcus pentosaceus*

**DOI:** 10.1186/s13213-021-01645-5

**Published:** 2021-08-31

**Authors:** Sadia Ahmed, Fatima Ashraf, Muhammad Tariq, Arsalan Zaidi

**Affiliations:** 1 National Probiotic Laboratory, National Institute for Biotechnology and Genetic Engineering College (NIBGE-C), Faisalabad, 38000 Pakistan; 2 Pakistan Institute of Engineering and Applied Sciences (PIEAS), Nilore, Islamabad, 45650 Pakistan

**Keywords:** Fermented cucumbers, Lactic acid bacteria, Functional properties, Inulin, Starter co-cultures

## Abstract

**Purpose:**

Cucumber fermentation is traditionally done using lactic acid bacteria. The involvement of probiotic cultures in food fermentation guarantees enhanced organoleptic properties and protects food from spoilage.

**Methods:**

Autochthonous lactic acid bacteria were isolated from spontaneously fermented cucumber and identified to species level. Only strains adjudged as safe for human consumption were examined for their technological and functional characteristics. Strain efficiency was based on maintaining high numbers of viable cells during simulated GIT conditions and fermentation, significant antioxidant activity, EPS production, nitrite degradation, and antimicrobial ability against Gram-positive and Gram-negative foodborne pathogens.

**Result:**

Two strains, *Lactiplantibacillus plantarum* NPL 1258 and *Pediococcus pentosaceus* NPL 1264, showing a suite of promising functional and technological attributes, were selected as a mixed-species starter for carrying out a controlled lactic acid fermentations of a native cucumber variety. This consortium showed a faster lactic acid-based acidification with more viable cells, at 4% NaCl and 0.2% inulin (w/v) relative to its constituent strains when tested individually. Sensory evaluation rated the lactofermented cucumber acceptable based on texture, taste, aroma, and aftertaste.

**Conclusion:**

The results suggest that the autochthonous LAB starter cultures can shorten the fermentation cycle and reduce pathogenic organism’ population, thus improving the shelf life and quality of fermented cucumber. The development of these new industrial starters would increase the competitiveness of production and open the country’s frontiers in the fermented vegetable market.

## Introduction

Raw fruits and vegetables constitute foods of high nutritional and functional value with fetching health-promoting effects (Güney and Güngörmüşler 2020). Cucumber (*Cucumis sativus*), primarily of Asian origin, has global appeal (Mukherjee et al. 2013). Because the vegetable’s juicy consistency renders it vulnerable to swift rot and short shelf life, considerable quantities are wasted, causing economic loss (Di Cagno et al. 2008). Fermentation offers an alternative route for prolonging its availability and transforming them into probiotic carriers handy for consumers with milk allergies and lactose intolerances (Karasu et al. 2010).

Fermented pickles are homemade products in most parts of the world, usually obtained by spontaneous fermentation (Zieliński et al. 2017). However, spontaneous fermentation being an uncontrolled, highly variable process necessitates (Sáez et al. 2018) the use of a controlled manufacturing process employing consortia of beneficial microbial autochthons for large-scale food making where sensory, nutritional, and technological attributes could be more consistently assured (Garmasheva et al. 2019). Autochthonous strains have the edge over allochthonous strains in being more niche-specific. Presently, no autochthonous lactic acid bacteria (LAB) starter is available for vegetable fermentation worldwide to give competitive high-quality products (Sáez et al. 2018). Therefore, finding LAB strains as autochthonous candidates for cucumber fermentation with dual function as bioprotective agents is highly prized. In contrast to the choices of LAB starters available for dairy, meat, and baked good fermentations, relatively few have been used for vegetable fermentations (Behera et al. 2020), with only a fraction of these have any purported probiotic potential (Guan et al. 2020).

LAB species such as the heterofermentative *Lactobacillus plantarum* and *L. pentosus* and the homofermentative *Pediococcus* spp. abound on the cucumber surfaces and present an opportunity to be assessed as a starter culture for controlled fermentation (Zhai et al. 2018). Mixed starter cultures are decidedly better in acidification and imparting flavors than monocultures (Nilchian et al. 2016). However, their ultimate use depends on the competition with the preexisting indigenous microbiota and the sensory attributes expected of the resulting products (Gardner et al. 2001). Ensuring good probiotic numbers in a fermented vegetable product can be achieved by mixing in prebiotics such as dietary fiber and cellulose or inulin (Güney and Güngörmüşler 2020). Historically, fermented vegetables have been a part of the diet of the Pakistani population. Despite this, there is a lack of commercially available autochthonous starters suitable for the fermentation of vegetables in the country. Vinegar-based pickling is the preferred mode of commercial manufacturing, which eliminates vegetable-associated lactic acid bacteria if any. Very few local studies have attempted looking at local LAB resources for potential use as starter cultures tailored for vegetable fermentation.

This work aims to collect LAB isolates from spontaneously LAB fermented cucumber, characterize and use autochthonous mixed starter strains to guarantee safety, functionality, and sensory properties of lacto-fermented cucumber.

## Materials and methods

### Leadoff micro-composition and selection

#### Isolation of LAB from lab-made spontaneously fermented (SF) cucumber

Fresh and tender cucumbers (organically and conventionally grown) were procured from the local fruit market of Islamabad and Faisalabad, respectively. Cucumbers were washed and cut into (approximately 2 × 7 cm) pieces, and every 200 g of these slices were dispensed into 500 mL airtight jars followed by the addition of 400 mL of the 3% (w/v) sterile brine solution. Jars were incubated at ambient temperature for fermentation of cucumber for 3 weeks. Natural fermentation was allowed on its own, depending on the naturally present microbes.

Fermented cucumbers (20 g) were blended with sterile saline solution (0.85% NaCl) in a stomacher (ProBlend Synbiosis, UK) for 2 min at high speed (400 strokes/min), and the cell suspension was removed from the stomacher bags. Cucumber cell suspension and brine samples were appropriately diluted in sterile saline solution (0.85% NaCl) and plated on De Man, Rogosa, and Sharpe (MRS) agar (Merck, Germany) supplemented with 0.05% L-cysteine (Oxoid, UK) to select for LAB. Presumptive LAB were isolated from spontaneously fermented cucumbers under aerobic and anaerobic conditions (Bactron-300, Shel Lab, USA) with 5% H_2_, 5% CO_2,_ and 90% N_2_. Plate Count Agar (PCA) (Merck, Germany), Oxytetra Glucose Yeast Agar Base (OGYE) (Himedia, India), and MacConkey Agar (LAB M Limited, UK) were used for the enumeration of total aerobic microbes, yeast and molds, and fecal coliform, respectively. *Streptococci* and *Lactococci* were cultured on KF Streptococcus Agar KFSA (Merck, Germany) and M17 Agar (LAB M Limited), and Reinforced Clostridial Agar (RCM) (Oxoid, UK) was used for anaerobically isolating Clostridial spp. (Montaño et al. 2004). Aerobic microbes such as yeasts and molds and fecal coliform organisms were checked using aerobic culturing conditions.

The isolates were chosen from different media plates based on distinct cell morphology, purified, and stored in 20% glycerol (v/v) at − 80 °C. The well-studied commercial probiotic strain *Lactiplantibacilllus plantarum* (ATCC 8014) (Huang et al. 2013), purchased from Microbiologics Inc., USA, was used as a reference strain.

#### Phenotypic and genotypic characterization

Bacterial isolates were Gram-stained, examined microscopically, and tested for catalase activity (Dash et al. 2012). Identification of the isolates was made by 16S rRNA gene sequencing. Following the manufacturer’s instructions, total bacterial DNA was isolated using a genomic DNA extraction kit (Thermo Scientific, Lithuania, European Union). The quality and concentration of DNA were assessed using a NanoDrop spectrophotometer (Thermo Scientific 2000C, Germany). The specific primer sets (Gene Link, USA) 357F (CCT ACG GGA GGC AGC AG) and 926R (CCG TCA ATT CMT TTR GT) were designed to amplify the V3–V5 regions of the 16S rRNA gene as previously reported (Sim et al. 2012). PCR products were sequenced on a BI3730XL 96-capillary DNA analyzer by Macrogen (Korea) using the same primer set as above. The sequences obtained were compared with the most recently released nonredundant DNA sequence database at the National Center for Biotechnology Information (NCBI) website http://www.ncbi.nlm.nih.gov/BLAST. All the identified sequences were deposited with NCBI, and their accession numbers were obtained. Multiple sequence analysis was done with ClustalW. Phylogenetic analyses were conducted using the Molecular Evolutionary Genetics Analysis (MEGA) version 10.0 software. Evolutionary histories were inferred using the maximum likelihood method with the Kimura 2-parameter model for 16S rRNA sequence analyses (Nel et al. 2020). The strengths of the internal branches of the resultant trees were statistically evaluated by bootstrap analysis with 100 bootstrap replications.

#### Selection of prospective probiotic cum starter candidates

Hemolysis and gelatinase activity was measured using a prescribed protocol (Kaktcham et al. 2018). A *Streptococcus pyogenes* strain (ATCC 19615^TM^) was used as a positive control for hemolysis (Songisepp et al. 2012) and *Bacillus cereus* (ATCC 1178) for gelatinase. The biogenic amine production of the LAB strains was measured in a medium consisting of decarboxylase agar (Himedia, India) having 2% (w/v) of one of the following: precursor amino acids L-histidine, L-ornithine (Scharlau, Spain), or L-tyrosine (Alfa Aesar, Germany) (Ji et al. 2013). Antibiotic susceptibility testing (AST) was performed using a commercial kit (E-Test, BioMérieux, France) according to recommendations of the European Food Safety Authority (EFSA), and strains were classified as resistant or susceptible, as previously reported (EFSA 2018). Strains were further screened for the presence of resistant genes using gene-specific primers *catA,* cat for chloramphenicol; *bla,* for ampicillin; *aadA, aadE,* and *ant(6)* for Streptomycin; *InuA* and *InuB*, for clindamycin; *tetM, tetK,* and *tetL* for tetracycline (Dec et al. 2017; Guo et al. 2017). The enzyme profiling was performed using a commercially available kit (API-ZYM, BioMérieux, France) according to the manufacturer’s instructions. A reference strain *Lactiplantibacillus plantarum* ATCC 8014, was additionally used.

### Probiotic potential of LAB strains

#### GIT persistence and colonization

Resistance to gastric acid and bile was determined using a published method (Jawan et al. 2019). Log-phase bacterial cultures were incubated in PBS at pH 1.5, 3.0 and in MRSc broth with porcine bile (Sigma-Aldrich, USA) for 3 h at 37 °C. Bacterial growth was determined by measuring OD_630 nm_ every 3 h for 9 h on a SpectraMax Plus 384 microplate reader. The phenol resistance of lactobacilli was quantified by inoculating log-phase bacterial cultures at an OD_630 nm_ of 0.1 into new MRS containing 0 to 0.4% w/v phenol (Merck, Germany) and measuring OD_630 nm_ every 30 min for 12 h using a SpectraMax Plus 384 microplate reader (Jawan et al. 2019). For prebiotic utilization ability, three commercially available prebiotics, namely Inulin (Alfa Aesar, Germany), Fructooligosaccharides (FOS), and Maltodextrin (Sigma-Aldrich, USA) were evaluated using an established protocol (Zago et al. 2011). Dextrose (Daejung, South Korea) was used as a positive control.

#### Effect of simulated GIT digestive process on strain viability

The protocol for *in vitro* digestion of bacterial cell suspensions in MRS broth and acidified cucumber brine and the recipe for GIT fluids were adapted from previously reported work (Weiss and Jespersen 2010). Briefly, log-phase cultures of test LAB strains were individually resuspended in acidified brine and MRS broth to an OD_630 nm_ of 0.6. About 2.4 mL of each of these bacterial suspensions was then separately added to 1 mL of fresh saliva solution and incubated for 5 min 37 °C. The simulated gastric digestion was initiated by mixing 6.4 mL of simulated gastric juice (pH 3.0) with salivary phase cellular resuspension and incubated for another 2 h at 37 °C. Finally, the 10 mL of these cell suspensions were then mixed with simulated duodenal juice (6 mL), bile solution (3.0 mL), and 1 M sodium bicarbonate solution (1.0 mL) (Merck, Germany) and again incubated for a further 2 h at 37 °C. All the digestion steps were performed under agitated conditions at 100 rpm (heating/cooling microplate shaker, VWR, USA). After simulated digestion, the mixture’s total cell viability was determined by plating on MRS media using a spiral plater and Q-count system (Advanced Instruments, USA). A method described by Zago et al. (2011) was used to perform the lysozyme resistance assay. The hydrophobic nature of the tested strains was measured using bacterial adhesion to hydrocarbons (BATH) protocol with xylene as solvent (Merck, Germany) and autoaggregation according to the methods previously described (Kaktcham et al. 2018).

#### Host-benefiting attributes

For coaggregation assay, logarithmic phase cultures of LAB strains grown in MRS broth at 37 °C and pathogen strains (*Escherichia coli* ATCC 25922, *S. pyogenes* ATCC 19615, *Staphylococcus aureus* ATCC 25923, *Pseudomonas aeruginosa* ATCC 15442, *Citrobacter freundii* ATCC 8090, and *B. cereus* ATCC 11778) grown in Nutrient broth (Oxoid, UK) also at 37 °C were used. The pathogen strains were all purchased from Microbiologics Inc., USA. Coaggregation was quantified as before (Kaktcham et al. 2018).

Six food-associated pathogenic species, namely, *E. coli* (ATCC 25922), *S. pyogenes* (ATCC 19615), *S. aureus* (ATCC 25923), *P. aeruginosa* (ATCC 15442), *C. freundii* (ATCC 8090), and *B. cereus (*ATCC 11778), were used to assess the antibacterial activity of the LAB strains. The LAB isolates were spotted onto MRS agar plates (1 μL, ~ 10^5^ cfu/mL, ~ 5 mm diameter) and incubated for 24 h and then overlaid with 0.8% (w/v) soft TSB agar premixed with 10^7^ cfu/mL of indicator strain. The plates were examined after 24 h of incubation for the presence of a zone of inhibition. The zone diameter of inhibition (ZDI) was measured and interpreted as strong when ZDI > 20 mm; 10–20 mm, intermediate; and weak when < 10 mm (Halder et al. 2017).

Two complementary methods (hydroxyl radical and superoxide anion scavenging) were performed to evaluate the antioxidant activity of the tested strains as reported (Ren et al. 2014). For evaluating BSH activity, LAB strains were tested using a plate assay method (Ji et al. 2013) on MRS agar medium supplemented with 0.37 g/L CaCl_2_ (Daejung, Korea) and 0.5% (w/v) different bile salts: sodium tauroglycocholate TGC, sodium taurodeoxycholate TDC (Himedia, India), sodium taurocholate TC (Chem-Impex International, Inc., USA), and sodium deoxycholate DC (Sigma-Aldrich, USA). The capacity to assimilate cholesterol was tested in MRS broth using 100 μg/mL water-soluble PEG-Cholesterol (Sigma Aldrich, USA) (Tomaro-Duchesneau et al. 2014).

Carbohydrate fermentation was determined with a Hi-Carbo Kit (Himedia, India). Log phase cultures of select strains were resuspended to a turbidity of 0.5 OD_600 nm_ and added to individual wells containing one of the 35 sugars and incubated at 37 °C for 24 and 48 h. Amylolytic, lipolytic, and phytase activities of potential starter cultures were determined aerobically and anaerobically (Taheri et al. 2009).

### Starter potential of LAB strains

The enzymes involved in anti-nutritional tannin and gallate metabolism were determined as described before (Sáez et al. 2018) with some modifications. The ability of LAB to deplete sodium nitrite was determined as described by Ren et al. (2014). EPS production of the isolates was confirmed by the method described before (Anandharaj et al. 2015). Total EPS (expressed as mg/L) was evaluated in each sample using glucose as standard (50–500 mg/L), and values were expressed as mean ± standard deviation of triplicates. The proteolytic activity of LAB strains was determined using the spectrophotometric assay described by Sáez et al. (2018). The results were expressed in mmol of free amino acids (FAA) per liter of milk by referring to a standard curve of L-leucine. Activities were classified as low, intermediate, and high (0–1, 1–2, and > 2 mmol/L, respectively). Tolerance to saline stresses was evaluated by assessing the growth of microorganisms in MRS broth supplemented with 2, 4, 7, or 10% (m/V) NaCl (Daejung, South Korea), respectively. Growth was determined through OD_600 nm_ increase measured at intervals during 24 h of incubation at 37 °C (Sáez et al. 2018).

#### Strain screening for compatibility in mixed consortia

Statistical differences among the isolates were pointed out through the Principal Components Analysis (PCA) done by the method given by Kumari et al. (2016). PCA makes it possible to distinguish between various potential Lactobacilli strains and identify the most promising starter culture. The relationship among the strains was determined by PCA using XLSTAT™ software. Eleven discriminating variables (acid and bile tolerance, hydrophobicity, auto and coaggregation, antimicrobial, EPS, proteolytic activity, antioxidant activity, cholesterol assimilation, and nitrite degradation) were assessed in 10 potential LAB strains. PCA was based on the model of varimax rotation.

The compatibility of selected starter strains was determined through agar diffusion and cross-streak assay (Sáez et al. 2018).

### Using autochthonous starters for lacto-fermentation of cucumber

Cucumber juice medium (CJM) was prepared as described elsewhere (Gardner et al. 2001) with a few modifications. Fresh organically grown cucumbers were blended using an automatic juice extractor (Black and Decker food factory FX1000, Turkey). The extracted juice was centrifuged (10,000 × *g*, 20 min, 4 °C), then filter-sterilized through a 0.22 μm filter (Millipore Corporation, Bedford, MA 01730, USA) and stored at − 20 °C before use. Select *L. plantarum* NPL 1258 and *P. pentosaceus* NPL 1264 were grown on MRS agar plates, and a colony of bacteria was transferred separately into filter-sterilized cucumber juices. The inoculated cucumber juice was incubated at 37 °C for 48 h until the inoculated juices were very turbid because of the growth of the inoculated bacterial cells.

Fresh and tender cucumbers (organic) were procured from the local fruit market of Islamabad and Faisalabad, respectively. Cucumbers (approximately 2 × 7 cm in length) were washed and blanched for 15 s at 80 °C (Reina et al. 2005) and cubed. Approximately 200 g of these cubes were dispensed into 500-mL airtight jars, followed by the addition of 400 mL of one of the following sterile brine solutions (4% w/v NaCl, pH 4):

- Control: no added bacterial culture
- A: with *L. plantarum* culture
- B: with 0.2% w/v inulin and *L. plantarum* culture
- C: with *P. pentosaceus* culture
- D: with 0.2% w/v inulin and *P. pentosaceus* culture
- E: with *L. plantarum* and *P. pentosaceus* cultures
- F: with 0.2% w/v inulin plus *L. plantarum* and our *P. pentosaceus* cultures

For the inoculum of starter culture, the bacteria were removed from the cucumber juice medium (CJM) by centrifugation at 3824 × *g*. The cell pellet was washed twice with saline solution and centrifuged. The final cell pellet was resuspended into an equal volume of saline solution. The jars were inoculated with the washed cells with an initial 107 cfu/mL population and were incubated at ambient temperature for fermentation.

#### Microbiological & biochemical analysis

The brines of the cucumber samples were analyzed during the period of fermentation. One milliliter of the sample was aseptically transferred to 9 mL of sterile saline solution, and appropriate dilution was poured on the MRS agar plates for LAB, and the nutrient agar was incubated at 37 °C for 24–48 h to determine the aerobic mesophilic bacteria. Yeast and molds were enumerated on OGYE media (Oxytetra Glucose Yeast Agar Base), fecal coliforms on MacConkey agar, and *Streptococci* and *Lactococci* on M17 agar (Montaño et al. 2004).

During fermentation, the pH of brine samples was regularly measured using a digital pH meter (model HI99161, Hanna Instruments, Germany), and the lactic and acetic acids produced in fermentation were measured using a commercial kit (Megazyme, USA).

#### Sensory analysis

Sensory evaluations of the fermented samples were carried out at the end of the process by a panel of 20 healthy individuals, all in the 25–35-year age bracket, half male and half female, all reasonably familiar with tasting pickles and Lacto-fermented products. Sensory attributes (taste, texture, crunchiness, saltiness, sharpness, aroma, flavor, color/appearance, and aftertaste) were evaluated using a 5-point hedonic scale (where 1 = dislike extremely and 5 = like extremely) (Güney and Güngörmüşler 2020). The panelists received samples distributed in randomly labeled transparent polypropylene cups that they could try once. The evaluation was repeated twice on two separate occasions. Sensory evaluation data were presented as means of the panelists’ scores. A standard *t* test was used to test for the statistical significance of the differences observed between the scores of the two tests.

### Statistical analysis

All samples were tested twice, and each experimental parameter was determined in triplicate. Results are expressed as mean ± SD. Statistical tests were one-way analyses of variance (ANOVA). When effects were significant (*P* < 0.05), Tukey’s test was used as a post hoc test. All statistical analyses were done using GraphPad Prism software (version 9 for Windows, GraphPad Software Inc, USA).

## Results

### Original microbiological profile of SF cucumber and choice of probiotic cum starter LAB

Forty-four bacterial isolates were obtained from lab-made spontaneously fermented cucumber (Table 1) and identified based on physiological, biochemical, and genotypic characteristics. Fermented cucumbers (organically grown) were enriched with *Lactobacillus* (46%), whereas *Enterococcus* (27%) were abundantly isolated from conventionally grown cucumbers. Five other genera were also frequently encountered in conventional-farmed fermented cucumbers, including *Pediococcus, Bacillus*, *Leuconostoc, Staphylococcus, and Citrobacter*.

**Table 1:** Background details of bacterial isolates from fermented cucumber

Portion of veg.	Fermented organic cucumber		Fermented non-organic cucumber	
	Aerobic	Anaerobic	Aerobic	Anaerobic
Brine	NPL 1277, *L. plantarum* ɷ	NPL 425, *L. plantarum* ɶ	NPL 1288, *E. faecalis* ɷ	NPL 1304, *E. faecium* ɶ
	NPL 1279, *L. plantarum* ɷ	NPL 427, *L. plantarum* ɶ	NPL 1289, *E. coli* ɷ	NPL 1286*, L. plantarum* ɶ
	NPL 1280, *L. plantarum* ɷ	NPL 428, *L. plantarum* ɶ	NPL 1290, *B*. *amyloliquefaciens* ɷ	NPL 1305, *L. pseudomesenteroides* ɶ
	NPL 1273, *S. epidermidis* ʨ	NPL 429, *L. plantarum* ɶ	NPL 1291, *P. pentosaceus* ɷ	NPL 1306, *L. plantarum* ɶ
		NPL 430, *L. plantarum* ɶ	NPL 1292, *E. hirae* ɷ	
		NPL 431, *L. plantarum* ɶ	NPL 1298, *E. cloacae* ʉ	
		NPL 432, *L. plantarum* ɶ	NPL 1299, *Enterobacter sp.* ʉ	
		NPL 433, *L. brevis* ɶ	NPL 1281, *B. contaminans* ʨ	
		NPL 434, *L. plantarum* ɶ	NPL 1295, *Enterococcus sp.* ʛ	
		NPL 436, *L. plantarum* ɶ	NPL 1296, *E. cloacae* ʛ	
		NPL 437, *L. plantarum* ɶ	NPL 1297, *E. cloacae* ʛ	
		NPL 1259, *L. plantarum* ɸ		
		NPL 1264, *P. pentosaceus* ɸ		
Pulp	NPL 1282, *B. halotolerans* ɷ	NPL 438, *L. plantarum* ɶ	NPL 1287, *K*. *pneumoniae* ɷ	NPL 1301, *E. faecium* ɸ
	NPL 1284, *L. plantarum* ʉ	NPL 440, *L. plantarum* ɶ	NPL 1300,*C. amalonaticus* ʉ	NPL 1302 , *E. hirae* ɸ
		NPL 441, *L. plantarum* ɶ		NPL 1303, *E. faecalis* ɸ
		NPL 442, *L. plantarum* ɶ NPL 443, *S. thermophilus* ɶ		
		NPL 1258, *L. plantarum* ɶ		

ɶ: MRS supplemented with L-cysteine

ɷ: Plate Count Agar (PCA)

ɸ: Reinforced Clostridial Agar (RCM)

ʉ: Oxytetra Glucose Yeast Agar Base (OGYE)

ʛ: KF Streptococcus agar KFSA

ʨ: MacConkey agar

**Fig. 1:**
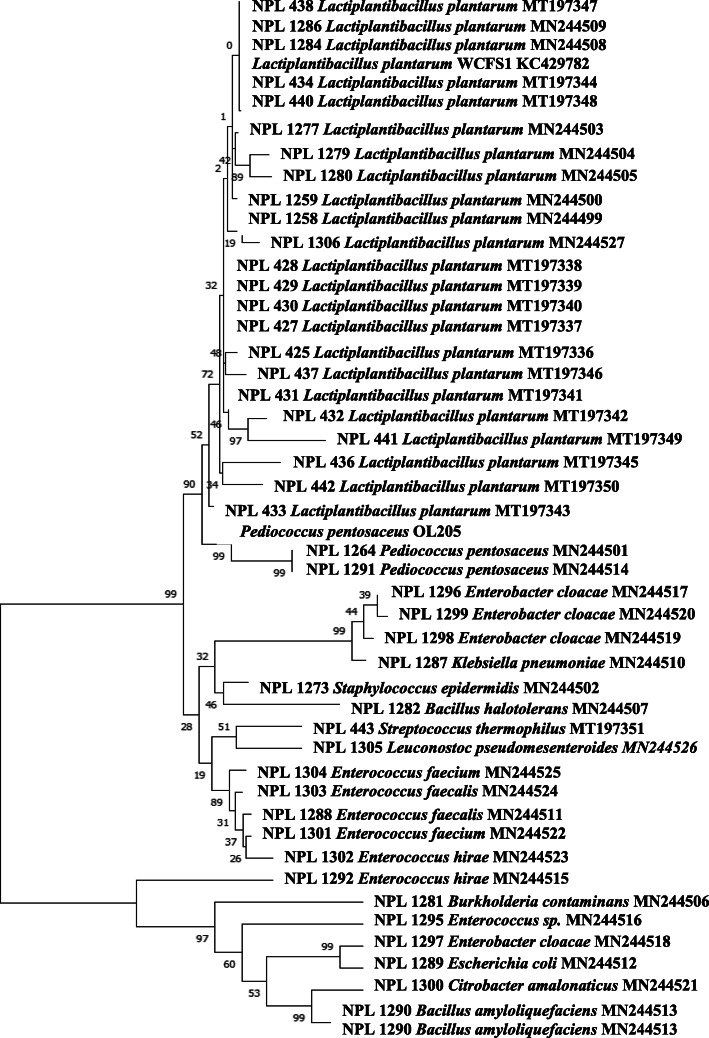
Molecular phylogenetic analysis. The maximum-likelihood method based on the Kimura 2-parameter model was used with 100 bootstrap replicates. Branches corresponding to partitions reproduced in less than 50% bootstrap replicates are collapsed. The percentage of replicate trees in which the associated taxa clustered together in the bootstrap test (1000 replicates) is shown next to the branches. Evolutionary analyses were performed in megaX

The 16s rDNA gene sequences of all isolates have been submitted to the Gene bank database, and the phylogenetic relationship between LAB was mapped based on the 16S rDNA sequences from evolutionary distances (Fig. 1). The maximum-likelihood method based on the Kimura 2-parameter model was used with 100 bootstraps in Molecular Evolutionary Genetics Analysis (MEGA) software, and three main clusters were identified. The first group included 27 strains of *L. plantarum*, and two strains of *P. pentosaceus* clustered together, followed by *Enterococcus* strains, whereas non-LAB strains clustered discretely from the LAB strains.

Strains of *E. faecium*, *E. faecalis,* and few strains of *L. plantarum* exhibited characteristics rendering them unsafe for human probiotic consumption. Both strains of *E. faecalis* were β hemolytic, gelatinase positive, and were resistant to aminoglycosides and tetracycline. All strains of *E. faecium* showed resistance to penicillin and aminoglycosides. Three of the *L. plantarum* strains were α hemolytic, six were gelatinase positive, three were found to produce biogenic amines, and three strains showed aminoglycosides and lincomycin resistance genes. The remaining ten *L. plantarum* strains and all *P. pentosaceus* were sensitive to antibiotics, did not produce biogenic amines, were non-hemolytic and gelatinase negative, thus deemed safe for use as starter cum probiotic inocula (Table 2).

**Table 2:** Safety assessment of the LAB strains

Strain code	Antibiotic susceptibility/ antibiotic resistance genes								Hemolytic activity	Gelatinase activity	Biogenic amine production			
	AM/*bla*	GM/*aac(6′)-aph(2″)*	KM/*aph(3′)-IIIa*	SM/(*aadA, aadE, ant(6))*	EM/*erm(A*), *erm(B*)	CM/*(InuA, InuB)*	TC/*(tetM, tetK, tetL)*	CL/*(catA,* cat)			His	Orn	Lys	Tyr
NPL 425	S/-	S/-	S/-	S/-	S/-	S/-	S/-	S/-	γ	+	-	-	-	-
NPL 427	S/-	S/-	S/-	S/-	S/-	S/-	S/-	S/-	γ	+	-	-	-	-
NPL 428	S/-	S/-	S/-	S/-	S/-	S/-	S/-	S/-	γ	+	-	-	-	-
NPL 429	S/-	S/-	S/-	S/-	S/-	S/-	S/-	S/-	γ	-	+	+	-	+
NPL 430	S/-	S/-	S/-	S/-	S/-	S/-	S/-	S/-	γ	+	-	-	-	-
NPL 431	S/-	R/+	S/-	R/+	S/-	S/-	S/-	R/+	γ	-	-	-	-	-
NPL 432	S/-	S/-	S/-	S/-	S/-	S/-	S/-	S/-	γ	+	-	+	-	-
NPL 433	S/-	S/-	S/-	S/-	S/-	S/-	S/-	S/-	γ	+	+	-	-	+
NPL 434	S/-	S/-	S/-	S/-	S/-	S/-	S/-	S/-	α	-	+	-	+	-
NPL 436	S/-	R/+	S/-	R/+	S/-	S/-	S/-	R/+	γ	-	-	-	+	-
NPL 437	S/-	S/-	S/-	S/-	S/-	S/-	S/-	S/-	α	-	-	-	+	+
NPL438	S/-	S/-	S/-	S/-	S/-	S/-	S/-	S/-	γ	-	+	+	-	+
NPL 440	S/-	S/-	S/-	S/-	S/-	S/-	S/-	S/-	α	-	-	+	-	-
NPL 441	S/-	R/+	S/-	R/+	S/-	S/-	S/-	R/+	γ	-	-	-	-	-
NPL 442	S/-	S/-	S/-	S/-	S/-	S/-	S/-	S/-	γ	-	+	+	-	+
NPL 443	S/-	S/-	S/-	R/+	S/-	S/-	R/+	S/-	γ	-	-	-		-
**NPL 1258**	S/-	S/-	S/-	S/-	S/-	S/-	S/-	S/-	γ	-	-	-	-	-
**NPL 1259**	S/-	S/-	S/-	S/-	S/-	S/-	S/-	S/-	γ	-	-	-	-	-
**NPL 1264**	S/-	S/-	S/-	S/-	S/-	S/-	S/-	S/-	γ	-	-	-	-	-
**NPL 1277**	S/-	S/-	S/-	S/-	S/-	S/-	S/-	S/-	γ	-	-	-	-	-
**NPL 1279**	S/-	S/-	S/-	S/-	S/-	S/-	S/-	S/-	γ	-	-	-	-	-
**NPL 1280**	S/-	S/-	S/-	S/-	S/-	S/-	S/-	S/-	γ	-	-	-	-	-
NPL 1282	S/-	S/-	S/-	S/-	S/-	S/-	S/-	S/-	α	+	-	-	-	-
**NPL 1284**	S/-	S/-	S/-	S/-	S/-	S/-	S/-	S/-	γ	-	-	-	-	-
**NPL 1286**	S/-	S/-	S/-	S/-	S/-	S/-	S/-	S/-	γ	-	-	-	-	-
NPL 1288	S/-	R/+	S/-	S/-	S/-	S/-	R/+	S/-	β	+	-	-	-	-
**NPL 1291**	S/-	S/-	S/-	S/-	S/-	S/-	S/-	S/-	γ	-	-	-	-	-
NPL 1301	R/+	S/-	S/-	R/+	S/-	S/-	S/-	S/-	γ	-	-	-	-	-
NPL 1303	S/-	R/+	S/-	S/-	S/-	S/-	R/+	S/-	β	+	-	-	-	-
NPL 1304	R/+	S/-	S/-	R/+	S/-	S/-	S/-	S/-	γ	-	-	-	-	-
NPL 1305	S/-	S/-	S/-	S/-	S/-	S/-	S/-	S/-	γ	-	+	-	+	-
**NPL 1306**	S/-	S/-	S/-	S/-	S/-	S/-	S/-	S/-	γ	-	-	-	-	-

*AM* ampicillin, *GM* gentamycin, *KM* kanamycin, *SM* streptomycin, *EM* erythromycin, *TC* tetracycline, *CM* clindamycin, *CL* chloramphenicol, *His* histidine, *Orn*, ornithine, *Lys* lysine, *Tyr* tyrosine. Results are means of three independent experiments with three repetitions ± SD (*n* = 3).

### Select *Lactobacillus* and *Pediococcus* strains demonstrate probiotic traits

#### LAB strains exhibit colonization and GIT persistence potential

None of our strains was found to be strongly hydrophobic, but three strains, NPL 1258, NPL 1279, and NPL 1280, belonging to *L. plantarum,* were moderately hydrophobic (< 70%). The rest exhibited low hydrophobicity (< 36%) (Table 3).

**Table 3:** Probiotic potential characteristics of the selected LAB strains

Strain code	pH tolerance (%)		Bile tolerance (%)		Hydrophobicity (%)	Autoaggregation (%)				NaCl tolerance (%)				Lysozyme resistance	Phenol resistance (0.4%)
	1.5	3	0.15	0.30	Xylene	***t***_**2**_	***t***_**4**_	***t***_**6**_	***t***_**24**_	2	4	7	10		
NPL 1258	45.07 ± 0.01	73.24 ± 0.1	70.22 ± 0.04	38.89 ± 0.03	54 ± 0.02	20 ± 0.02	32 ± 0.01	46 ± 0.12	87 ± 0.01	+	+	+	-	+	+
NPL 1259	49.86 ± 0.03	62.43 ± 0.02	62.35 ± 0.03	27.41 ± 0.12	27 ± 0.01	11 ± 0.01	15 ± 0.01	18 ± 0.02	25 ± 0.01	+	+	+	-	+	+
NPL 1264	44.74 ± 0.05	67.01 ± 0.03	73.33 ± 0.02	38.18 ± 0.04	30 ± 0.04	13 ± 0.03	19 ± 0.04	28 ± 0.03	33 ± 0.11	+	+	+	-	+	+
NPL 1277	46.67 ± 0.01	73.33 ± 0.05	49.02 ± 0.01	13.73 ± 0.03	22 ± 0.0	28 ± 0.0	36 ± 0.0	50 ± 0.04	88 ± 0.03	+	+	-	-	+	+
NPL 1279	20.18 ± 0.04	46.49 ± 0.03	43.31 ± 0.06	19.75 ± 0.07	41 ± 0.11	34 ± 0.01	39 ± 0.02	43 ± 0.03	72 ± 0.05	+	+	-	-	+	+
NPL 1280	16.67 ± 0.02	25 ± 0.05	46.36 ± 0.07	23.84 ± 0.02	45 ± 0.06	10 ± 0.03	17 ± 0.01	26 ± 0.02	59 ± 0.06	+	+	-	-	+	+
NPL 1284	25 ± 0.01	31.25 ± 0.02	31.25 ± 0.03	13.75 ± 0.04	14 ± 0.05	27 ± 0.04	39 ± 0.04	53 ± 0.06	99 ± 0.01	+	+	+	-	+	+
NPL 1286	33 ± 0.03	87 ± 0.05	27.27 ± 0.02	19.39 ± 0.01	16 ± 0.02	12 ± 0.03	24 ± 0.05	37 ± 0.03	98 ± 0.00	+	+	+	-	+	+
NPL 1291	33.68 ± 0.02	51.58 ± 0.06	57.58 ± 0.01	34.09 ± 0.03	34 ±0.01	10 ± 0.02	22 ± 0.01	33 ± 0.0	67 ± 0.02	+	+	+	-	+	+
NPL 1306	30.83 ± 0.03	34.72 ± 0.03	59.64 ± 0.02	13.36 ± 0.02	31 ± 0.0	12 ± 0.01	19 ± 0.02	38 ± 0.02	65 ± 0.01	+	+	-	-	+	+
ATCC 8014	41.02 ± 0.01	66.31 ± 0.08	61.21 ± 0.01	25.42 ± 0.03	35 ± 0.11	17 ± 0.03	36 ± 0.02	41 ± 0.02	54 ± 0.01	+	+	-	-	+	+

Results are means of three independent experiments with three repetitions ± SD (*n* = 3).

Auto-aggregation results of test strains were highly variable. Auto-aggregation of LAB strains increased with the increase of incubation time (Table 3). Among the *L. plantarum* strains, NPL 1258, NPL 1277, NPL 1284, and NPL 1286 exhibited the highest auto-aggregation (85–100%) after 24 h of incubation. In contrast, other strains showed moderate auto-aggregation (20–50%).

The growth of *L. plantarum* (NPL 1258, NPL 1259, NPL 1286) and *P. pentosaceus* (NPL 1264) was markedly less at pH 1.5 than at pH 3.0 (Table 3). All strains of *L. plantarum* except for NPL 1286 could well tolerate the exposure to 0.15% (w/v) porcine bile. However, the survival rate was found to be low for *L. plantarum* strains NPL 1286. All the strains of *L. plantarum* and *P. pentosaceus* strains were also tolerant to phenol and lysozyme.

**Fig. 2:**
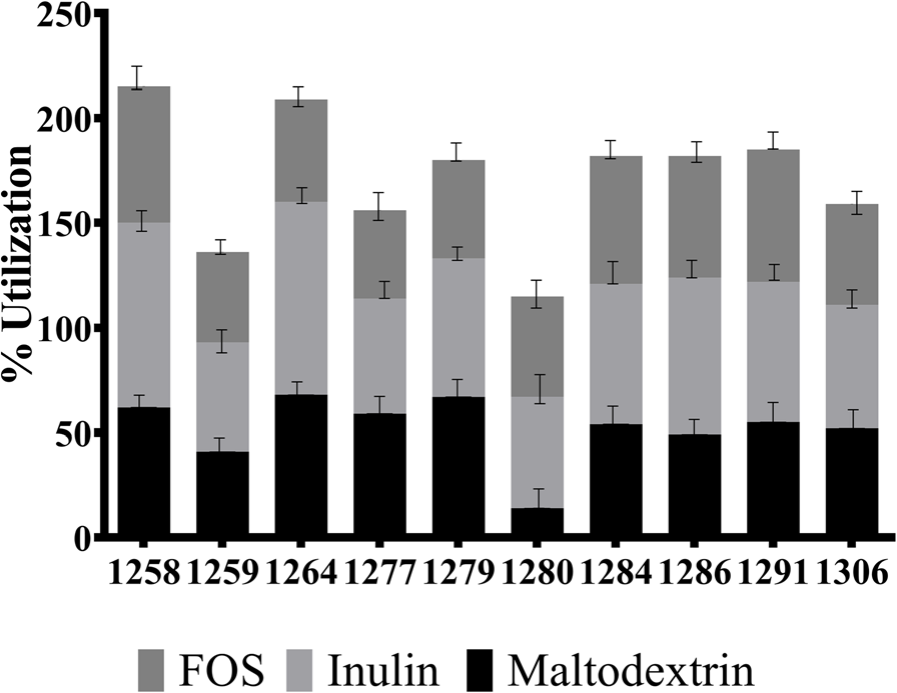
Prebiotic utilization by LAB strains during growth. 1258: *L. plantarum*, 1259: *L. plantarum,* 1264: *P. pentosaceus*, 1277: *L. plantarum*, 1280: *L. plantarum*, 1284: *L. plantarum,* 1286: *L. plantarum,* 1291: *P. pentosaceus*, 1301: *L. plantarum*. Results are means of three independent experiments with three repetitions ± SD, *n* = 3

All the strains grew strongly in the presence of inulin (achieving 80% growth on glucose) (Fig. 2). *L. plantarum* strains NPL 1258 and NPL 1286 and *P. pentosaceus* strain NPL 1291 utilized inulin the most (84%, 85%, and 71%, respectively). Fructo-oligosaccharide utilization was observed in NPL 1291, NPL 1284, and NPL 1258. Some of the test strains were poorly fermentative of maltodextrin.

#### LAB strains exhibit good tolerance of simulated human digestion

**Fig. 3:**
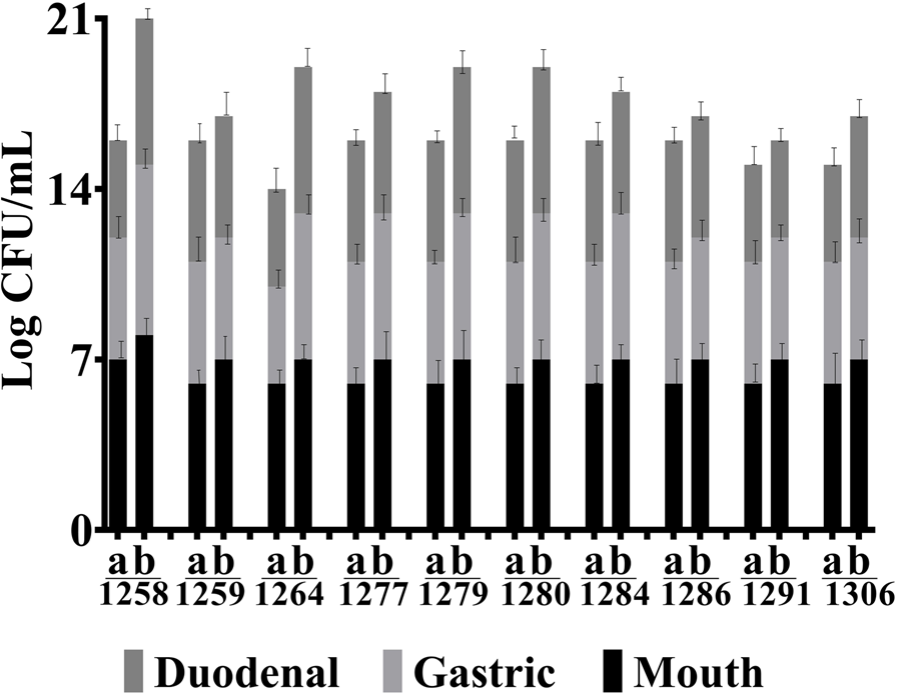
*In vitro* digestion assay of the selected Lactobacilli without (**a**) and with (**b**) food matrix. 1258: *L. plantarum*, 1259: *L. plantarum,* 1264: *P. pentosaceus*, 1277: *L. plantarum*, 1280: *L. plantarum*, 1284: *L. plantarum,* 1286: *L. plantarum,* 1291: *P. pentosaceus*, 1301: *L. plantarum*. Results are means of three independent experiments with three repetitions ± SD, *n* = 3

Food matrix effectively buffered tested LAB strains against simulated digestive fluids (Fig. 3). The simulated gastric fluid being more deleterious (1–1.5 log decrease) than simulated duodenal fluids (0.5–1.0 log decrease). The buffering effect of the vegetable matter was most pronounced for *L. plantarum* strain NPL 1258 and *P. pentosaceus* strain NPL 1264 which registered a nonsignificant decrease in number on exposure to simulated digestive fluids, whereas the *L. plantarum* strains NPL 1279 and NPL 1306 were most vulnerable to digestive action irrespective of whether they are enclosed in a vegetable matrix or not.

#### Select LAB exhibit promising host-benefiting traits

The antimicrobial spectrum of all LAB strains against six pathogenic bacteria was demonstrated by the agar overlay method. Some strains of *L. plantarum* variably inhibited the growth of pathogen indicators (Table 4). Among the *L. plantarum* strains NPL 1258 and NPL 1259 and *P. pentosaceus* strain NPL 1280 were most significantly antagonistic. Table 5 showed that all the strains could co-aggregate with the pathogens tested, although the magnitude varied from strain to strain. *L. plantarum* strains NPL 1258 and *P. pentosaceus* NPL 1264 exhibited the highest coaggregation with all pathogens tested (63–97%) following a 4-h incubation period.

**Table 4:** Functional properties of selected LAB strains

Strain code	Diameter of inhibition zone (mm)^*^						Deconjugation of bile salts^**ɷ**^			Decarboxylation of phenolics ^**ɣ**^	
	***S. p***	***S. a***	***E. c***	***B. c***	***P. a***	***C. f***	TGC	TC	TDC	Tann	Gall
NPL 1258	+++	+++	+++	++	++	++	+	+	+	+	+
NPL 1259	++	++	++	++	+++	++	+	+	-	+	+
NPL 1264	+++	++	+++	++	+++	++	+	+	-	+	+
NPL 1277	++	++	+++	++	++	++	-	+	-	+	+
NPL 1279	+++	++	++	++	++	++	+	+	-	+	+
NPL 1280	+	+	+++	+	+	+	+	+	-	+	+
NPL 1284	+++	+++	+++	++	++	++	+	+	+	+	+
NPL 1286	++	++	++	++	++	++	+	+	-	+	+
NPL 1291	+	+	++	++	+	++	+	+	-	+	+
NPL 1306	++	+++	++	++	+++	++	+	+	-	+	+
ATCC 8014	++	++	++	++	++	++	+	+	-	+	+

*S. p*, *Streptococcus pyogenes* ATCC 19615; *S. a, Staphylococcus aureus* ATCC 25923, *E. c, Escherichia coli* ATCC 25922; *B. c, Bacillus cereus* ATCC 11778; *P. a. Pseudomonas aeruginosa* ATCC 15442; *C. f. Citrobacter freundii* ATCC 8090

* (+) weak (< 10 mm), (++) intermediate (10–20 mm), (+++) strong (< 20 mm), (−) no zone

^ɷ^ *TGC*, tauroglycocholate; *TC*, sodium taurocholate; *TDC*, sodium taurodeoxycholate; *DC*, sodium deoxycholate

^ɣ^ (+) present, (-) absent. Results are means of three independent experiments with three repetitions ± SD (*n* = 3)

**Table 5:** Functional properties of selected LAB strains

Strain code	Antioxidant activity		Cholesterol assimilation (%)	Nitrite degradation (%)	Proteolytic activity (mmol/L)	EPS (mg/L)	Coaggregation (%)				
	Hydroxyl radical (%)	Superoxide anions (%)					***S. a***	***S. p***	***E. c***	***B. c***	***P. a***
NPL 1258	84 ± 0.22	64.65 ± 0.17	44 ± 0.12	75 ±0.01	1.89 ± 0.11	85.5 ± 0.01	77 ± 0.11	85 ± 0.05	66 ± 0.02	52 ± 0.01	52 ± 0.01
NPL 1259	48 ± 0.12	85.5 ± 0.22	38 ± 0.02	59 ± 0.12	1.73 ± 0.0	74.9 ± 0.12	31 ± 0.03	43 ± 0.08	87 ± 0.04	41 ± 0.03	41 ± 0.03
NPL 1264	73 ± 0.14	64.9 ± 0.32	33 ± 0.11	69 ± 0.05	0.76 ± 0.0	92.1 ± 0.15	97 ± 0.06	66 ± 0.13	53 ± 0.04	63 ± 0.04	63 ± 0.04
NPL 1277	52 ± 0.31	46.15 ± 0.45	22 ± 0.21	36 ± 0.03	1.61 ± 0.16	86.8 ± 0.17	34 ± 0.13	82 ± 0.16	62 ± 0.03	47 ± 0.5	47 ± 0.5
NPL 1279	60 ± 0.11	76.85 ± 0.27	12 ± 0.12	55 ± 0.17	1.38 ± 0.09	88.6 ± 0.21	74 ± 0.16	44 ± 0.04	36 ± 0.11	54 ± 0.11	54 ± 0.11
NPL 1280	56 ± 0.16	68.65 ± 0.22	29 ± 0.05	65 ± 0.02	1.41 ± 0.05	76.1 ± 0.33	87 ± 0.25	76 ± 0.02	55 ± 0.03	48 ± 0.21	48 ± 0.21
NPL 1284	74 ± 0.15	76.31 ± 0.13	31 ± 0.02	56 ± 0.14	1.60 ± 0.02	85.2 ± 0.12	29 ± 0.15	22 ± 0.05	48 ± 0.02	63 ± 0.04	63 ± 0.04
NPL 1286	66 ± 0.06	84.18 ± 0.17	23 ± 0.16	18 ± 0.11	1.67 ± 0.11	71.9 ± 0.11	43 ± 0.01	52 ± 0.11	58 ± 0.01	33 ± 0.04	33 ± 0.04
NPL 1291	53 ± 0.11	64.98 ± 0.19	19 ± 0.41	58 ± 0.04	0.55 ± 0.02	88.91 ± 0.02	63 ± 0.00	73 ± 0.05	61 ± 0.01	16 ± 0.2	16 ± 0.2
NPL 1306	38 ± 0.09	44.34 ± 0.32	20 ± 0.01	34 ± 0.01	1.52 ± 0.03	91 ± 0.04	75.3 ± 0.02	58 ± 0.21	49 ± 0.12	82 ± 0.01	64 ± 0.01
ATCC 8014	44 ± 0.03	62.34 ± 0.02	25 ± 0.04	56 ± 0.02	0.92 ± 0.01	83 ± 0.01	66.78 ±0.2	67 ± 0.11	56 ± 0.11	71 ± 0.01	39 ± 0.02

*S. p*, *Streptococcus pyogenes* ATCC 19615; *S. a*, *Staphylococcus aureus* ATCC 25923, *E. c, Escherichia coli* ATCC 25922, *B. c Bacillus cereus* ATCC 11778. *P. a Pseudomonas aeruginosa* ATCC 15442. Results are means of three independent experiments with three repetitions ± SD (*n* = 3).

All strains demonstrated deconjugation with sodium tauroglycocholate (TGC) and sodium taurocholate (TC) salts with varying substrate affinity. In general, most strains showed the least preference towards deconjugation of sodium taurodeoxycholate (TDC) compared with other bile salts (Table4).

Cholesterol concentrations in the cell culture medium decreased after the initial solution was co-cultured with all the *Lactobacillus* strains (Table 5). Among the tested strains, NPL 1258 exhibited the highest cholesterol removal rates (44%). The strains NPL 1279 and NPL 1291 showed weaker rates of cholesterol removal (12% and 19%, respectively).

All *Lactobacillus* strains exhibited antioxidative activity (Table 5). The scavenging rates for hydroxyl radicals ranged from 38 to 84%, and those for superoxide anion radicals ranged from 44 to 85.5%. NPL 1258 strain showed the highest capacity to scavenge hydroxyl radicals (84%), and NPL 1259 showed the highest capacity to scavenge superoxide anion radicals (85.5%).

*L. plantarum* strains NPL 1258, NPL 1284, and NPL 1286 and *P. pentosaceus* strain NPL 1264 can utilize a multitude of sugar moieties such as monosaccharides, oligosaccharides, and some trisaccharides (raffinose), along with some sugar alcohols (sorbitol, xylitol, and mannitol) and glycosides (esculin and salicin). Our strains are unable to metabolize disaccharides (maltose, lactose, and melibiose) and deoxy sugars (fucose and rhamnose) (Table 6). Malonate utilization seems to be absent in all *L. plantarum* and *P. pentosaceus* strains.

**Table 6:** Enzyme characterization and carbohydrate utilization of selected LAB

		NPL 1258	NPL 1259	NPL 1264	NPL 1277	NPL 1279	NPL 1280	NPL 1284	NPL 1286	NPL 1291	NPL 1306	ATCC 8014
**Sr. no.**	**Carbohydrate fermentation**											
1	Lactose	+	+	+	+	+	+	+	+	+	+	+
2	Xylose	+	+	+	+	+	+	+	+	+	+	+
3	Maltose	+	+	-	+	+	+	+	+	-	+	+
4	Fructose	+	-	+	+	-	+	+	+	+	+	+
5	Dextrose	+	-	+	+	-	+	+	-	+	+	+
6	Galactose	+	-	+	+	-	+	+	-	+	+	+
7	Raffinose	+	-	+	+	-	+	+	+	+	-	-
8	Trehalose	+	+	+	+	+	+	+	+	+	+	+
9	Melibiose	-	-	-	-	-	-	-	-	-	-	-
10	Sucrose	+	-	+	+	-	+	+	+	+	+	+
11	L-Arabinose	+	+	+	+	+	+	+	+	+	+	+
12	Mannose	+	-	+	+	-	+	+	+	+	+	+
13	Inulin	+	+	+	+	+	+	+	+	+	+	+
14	Sodium gluconate	-	-	-	-	-	-	-	-	-	-	-
15	Glycerol	-	-	-	-	-	+	+	-	-	-	-
16	Salicin	+	+	+	+	+	+	+	+	+	-	+
17	Dulcitol	-	-	-	-	-	-	+	-	-	-	-
18	Inositol	-	-	-	-	-	-	-	-	-	-	-
19	Sorbitol	+	+	+	+	+	+	+	+	+	+	+
20	Mannitol	+	+	+	+	+	+	+	+	+	+	+
21	Adonitol	-	-	-	-	-	-	+	-	-	-	+
22	Arabitol	-	-	-	-	-	-	+	-	-	+	+
23	Erythritol	-	-	-	-	-	-	-	-	-	-	-
24	α-Methyl-D glucoside	-	-	-	-	-	-	+	-	-	-	-
25	Rhamnose	-	-	-	-	-	-	+	-	-	-	-
26	Cellobiose	+	+	+	+	+	+	+	+	+	+	+
27	Melezitose	-	+	-	-	-	-	+	-	-	-	-
28	α-methyl-D mannoside	+	+	-	-	-	-	+	-	-	-	-
29	Xylitol	+	+	-	-	-	-	+	-	-	-	-
30	ONPG	+	+	-	-	-	-	+	-	-	-	-
31	Esculin hydrolysis	+	+	+	+	+	+	+	+	+	+	+
32	D-Arabinose	+	+	+	+	+	+	+	+	+	+	+
33	Citrate utilization	-	+	-	-	-	+	+	+	-	-	+
34	Malonate utilization	-	+	-	-	-	-	-	-	-	-	-
35	Sorbose	-	-	-	-	-	-	-	-	-	-	-
**Sr. no.**	**Enzyme activity**											
1	Alkaline phosphatase	0	0	0	0	0	0	0	0	0	0	0
2	Esterase (C 4)	0	0	0	0	0	0	0	0	0	0	0
3	Esterase lipase (C 8)	5	5	10	5	10	10	10	5	10	5	5
4	Lipase (C 14)	0	0	0	0	0	0	0	0	0	0	0
5	Leucine arylamidase	> 40	20	20	20	> 40	> 40	> 40	20	10	10	> 40
6	Valine arylamidase	20	20	20	20	20	20	20	20	5	5	10
7	Cystine arylamidase	> 40	> 40	20	20	20	20	20	20	20	20	20
8	Trypsin	0	0	0	0	0	0	0	0	0	0	0
9	α-chymotrypsin	0	0	0	0	0	0	0	0	0	0	0
10	Acid phosphatase	5	5	5	5	5	5	5	5	5	5	10
11	Naphthol-AS-BI-phosphohydrolase	5	5	5	5	5	5	5	5	5	5	20
12	α-galactosidase	0	0	0	0	0	0	0	0	0	0	0
13	ß-galactosidase	20	20	> 40	20	20	> 40	20	20	20	20	20
14	ß-glucuronidase	0	0	0	0	0	0	0	0	0	0	0
15	α-glucosidase	0	0	0	0	0	0	0	0	0	0	0
16	ß-glucosidase	20	10	20	10	5	5	5	10	5	10	20
17	N-acetyl-ß-glucosaminidase	0	0	0	0	0	0	0	0	0	0	0
18	α-mannosidase	0	0	0	0	0	0	0	0	0	0	0
19	α-fucosidase	0	0	0	0	0	0	0	0	0	0	0

Enzyme activities were assessed through API-ZYM galleries that were expressed in terms of color intensity, which ranged from 0 (no activity), 5–10 (low), 30 (moderate), and ≥ 40 nmol (strong) of substrate hydrolyzed following 4 h incubation at 37 °C. Color intensity was judged with reference to an API ZYM color chart provided by the manufacturer. ‘+’ shows a positive reaction; ‘−’ shows a negative reaction

The select *L. plantarum* strains and *P. pentosaceus* exhibited peptidase and esterase lipase (C8) activities. Alkaline phosphatase, esterase lipase (C4, C14), trypsin, and α-mannosidase and α-fucosidase were not active in the selected strains, nor activities of enzymes such as α-chymotrypsin, α-galactosidase, β-glucuronidase, N-acetyl-β-glucosaminidase, N-acetyl-β-glucosaminidase activities deemed undesirable were recorded (Table 6). None of the strains isolated in this study showed amylolytic, lipolytic, or phytase activities (data not shown).

#### LAB strains display good starter aptitude

All selected *L. plantarum* strains and *P. pentosaceus* strains were positive for tannase and gallate decarboxylase activities (Table 4). In this research, all strains used could produce EPS ranging from 88.91 to 193.7 mg/L (Table 5). Among them, *L. plantarum* NPL 1258 produced copious amounts of EPS, 193.7 mg/L. EPS production was the least in *P. pentosaceus* strain NPL 1291 (88.91 mg/L). Moreover, all the *L. plantarum* strains displayed intermediate proteolytic activity (FAA 1 to 2 mmol/L), whereas *P*. *pentosaceus* strains exhibited low proteolytic activity (FAA < 1 mmol/L). The maximum proteolytic activity value (1.89 ± 0.11 mmol/L) was found for *L. plantarum* NPL 1258, whereas the minimum value (0.55 ± 0.02 mmol/L) was seen in *P. pentosaceus* strain NPL 1291 (Table 5).

All the tested strains can reduce the nitrite concentration (Table 5). The highest sodium nitrite depletion rates were exhibited by the *L. plantarum* strains NPL 1258 (75%), *P. pentosaceus* NPL 1264 (69%), followed by *L. plantarum* NPL 1284 (65%). However, the lowest degradation rates were observed for *L. plantarum,* NPL 1286 (18%). These results show that most of the tested strains are highly effective in depleting sodium nitrite.

Growth in 2% NaCl was interpreted as strong when it approximated average growth (without NaCl), which was true for several strains (6 out of 10) (Table 3). Growth in the presence of 4% NaCl was typically halved by 50%. However, several strains of *L. plantarum* exhibited detectable growth at concentrations as high as 7% NaCl. In the present study, *L. plantarum* strain NPL 1259 was most robustly saline tolerant, withstanding 2%, 4%, and 7% NaCl by achieving 81%, 72%, and 65 % respectively of a typical growth profile.

#### Multivariate analysis of the most suitable starter culture

**Fig. 4:**
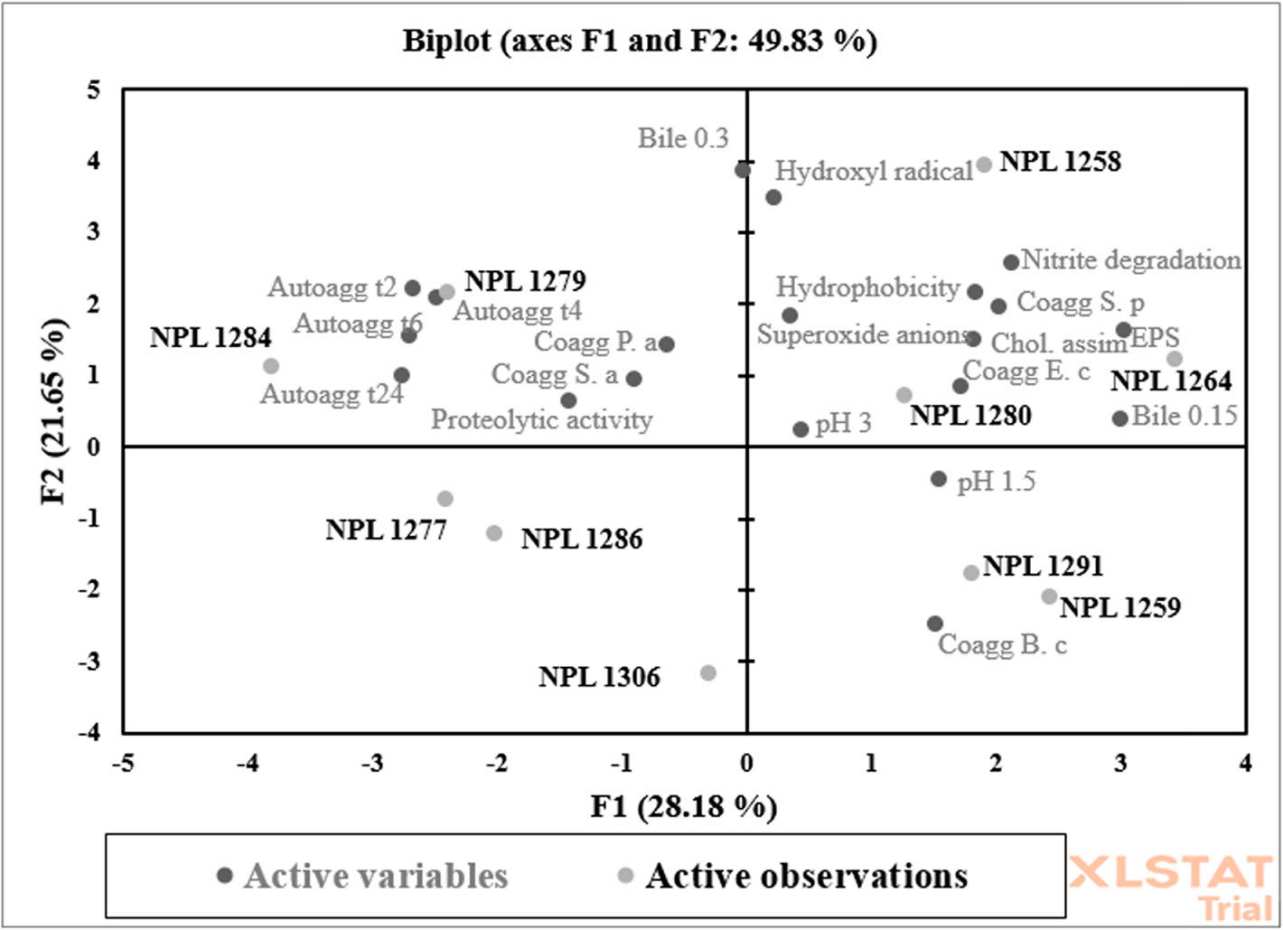
PCA biplot projecting the probiotic potential starter culture variables and strains

Principal component analysis was used to single out the most promising starter culture for carrying out directed Lacto fermentation of cucumber. The biplot graphs on PCA analysis are presented in Fig. 4. The first two factors represented 49.83% of the variability. From PCA analysis, *L. plantarum* strains NPL 1258 and NPL 1280 and *P. pentosaceus* strain NPL 1264 were more associated with starter culture and probiotic potential characteristics selected as a suitable candidate for lactic acid fermentation of cucumber. These three strains were further tested for their compatibility with each other for mixed culture through agar diffusion assay and cross-streak assay. No inhibition halos of *L. plantarum* strain NPL 1258 cell-free supernatants against the *P. pentosaceus* NPL 1264 were observable, suggesting the absence of antimicrobial substances that could prevent using these strains in mixed cultures. However, the limited growth of *L. plantarum* strains NPL 1280 was observed against *P. pentosaceus* NPL 1264. The cross-streak assay showed similar results, as no evident competition was noticeable at sites of co-growth in a solid medium in combination with NPL 1258 and NPL 1264, allowing their potential use as a mixed starter (results not shown).

### Lacto-fermentation of cucumber using select starter strains

#### *Lactiplantibacilllus plantarum* and *Pediococcus pentosaceus* driven cucumber fermentation prevents spoilage

**Fig. 5:**
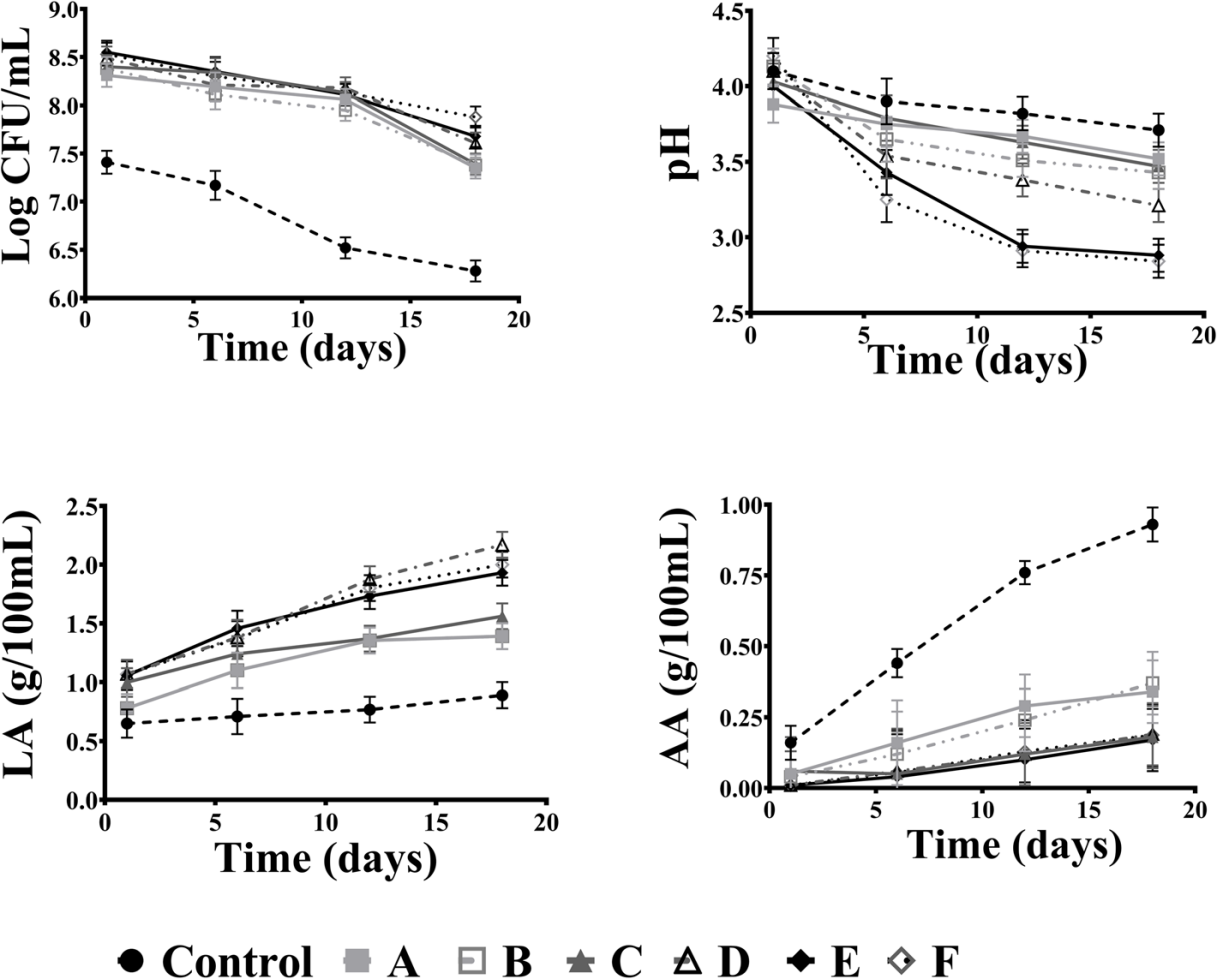
Microbiological and physicochemical analysis of lacto-fermented cucumber during fermentation period. **a** Changes in viable cell number of LAB in different samples, **b** pH, **c** LA (Lactic acid), and **d** AA (Acetic acid) during fermentation. Control: without bacterial inocula with 4% (w/v) NaCl, pH 4; A: *L. plantarum* NPL 1258 with 4% (w/v) NaCl, pH 4; B: *L. plantarum* NPL 1258 with 4% (w/v) NaCl, 0.2% inulin, pH 4; C: *P. pentosaceus* NPL 1264 with 4% (w/v) NaCl, pH 4; D: *P. pentosaceus* NPL 1264 with 4% (w/v) NaCl, 0.2% inulin, pH 4; E: *L. plantarum* NPL 1258 and *P. pentosaceus* NPL 1264 with 4% (w/v) NaCl, pH 4; F: *L. plantarum* NPL 1258 and *P. pentosaceus* NPL 1264 with 4% (w/v) NaCl, 0.2% inulin, pH 4. Results are means of three independent experiments with three repetitions ± SD, *n* = 3

Plating count determined the microbial changes in different groups during the fermentation. In all controlled fermented cucumber samples, LAB strains were the prevailing microorganisms throughout the process, and the maximum population of the inoculated strain was in treatment F (Fig. 5). The initial salt and inulin concentrations of 4% (w/v) and 0.2% respectively in the treated sample favored rapid growth of mixed strains. LAB population was 8.3 log10 cfu/mL on the sixth day of fermentation which only reduced slightly by the 18th day. Whereas in other treatments, bacterial viability was significantly reduced to 7 log10 cfu/mL till the 18th day of fermentation. Yeast and other pathogenic bacteria were observed in the control group after the sixth day. The number of LAB is also significantly reduced after the 6th day of fermentation.

#### Defined lacto-fermentation of cucumber driven by the high lactic acid content

On the first day of fermentation, the pH value of fermented samples ranged from 3.9 to 4.4. Following 18 days of fermentation, the pH of the brine solutions decreased from 3.5 to 3 (Fig. 5). The pH drop in all treatments was highly significant (*P* < 0.05) between the control sample and others, but there were no significant differences among treatments during fermentation. The pH drop was sharpest in treatments E and F and then plateauing to levels like others. The decrease of pH values in A and B samples were slighter than others. Lactic acid (g/100 mL) production continually increased in all inoculated samples relative to the control. Acetic acid (g/100 mL) production was negligible in all treatment samples except the control sample, where it spiked at the end.

#### Sensory acceptability of lacto-fermented cucumber

**Fig. 6:**
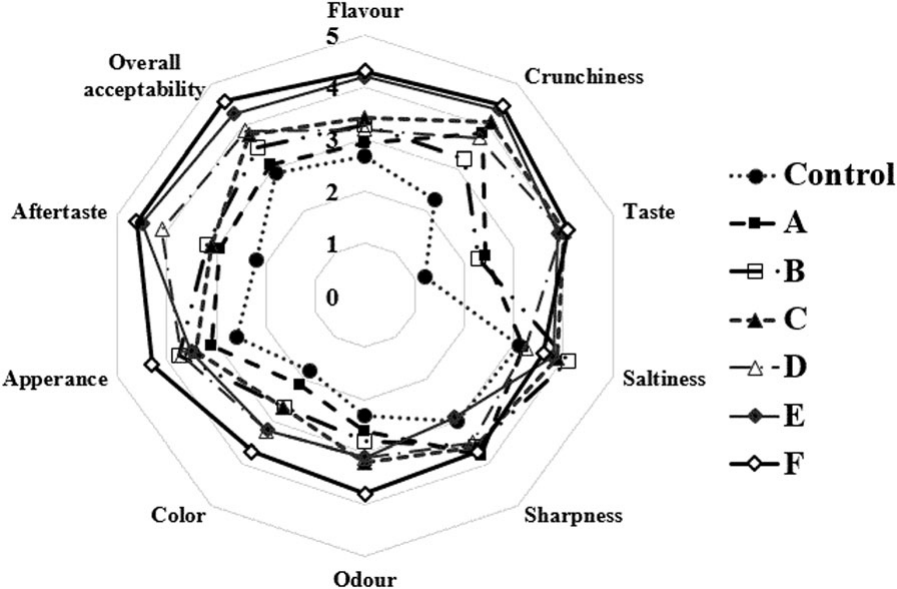
Sensory analysis of lacto-fermented cucumber after fermentation period. Control: without bacterial inocula with 4% (w/v) NaCl, pH 4; A: *L. plantarum* NPL 1258 with 4% (w/v) NaCl, pH 4; B: *L. plantarum* NPL 1258 with 4% (w/v) NaCl, 0.2% inulin, pH 4; C: *P. pentosaceus* NPL 1264 with 4% (w/v) NaCl, pH 4; D: *P. pentosaceus* NPL 1264 with 4% (w/v) NaCl, 0.2% inulin, pH 4; E: *L. plantarum* NPL 1258 and *P. pentosaceus* NPL 1264 with 4% (w/v) NaCl, pH 4; F: *L. plantarum* NPL 1258 and *P. pentosaceus* NPL 1264 with 4% (w/v) NaCl, 0.2% inulin, pH 4. Results are means of three independent experiments with three repetitions ± SD, *n* = 3

Sensory evaluation of fermented cucumber samples was performed at the culmination of the process using a panel of 10 non-trained persons. For flavor, the panelists gave an overall higher rank to samples made using both candidate LAB starter strains plus inulin than all other treatments (Fig. 6). The crunchiness was best appreciated in products made using dual than mono starters. LAB strains invariably contribute to the aroma, texture, and flavor of fermented products. The sharp increase in acidity minimized the influence of spoilage bacteria and consistently improved the microbiological and sensory quality of the fermented product than would be possible in fermentations without defined starters (Tamang and Tamang 2010).

## Discussion

Fermented cucumber is a popular culinary choice in many Asian countries (Behera et al. 2020). Fermented vegetables containing LAB are being increasingly researched because of their benefits to vegetarians and individuals with dairy intolerances (Di Cagno et al. 2013). Many vegetables with good nutritional content but otherwise poor organoleptics can be rendered flavorful and desirable for consumption when fermented by LAB species (Alan et al. 2018). Autochthonous LAB abounds spontaneously fermentation vegetables, an essentially stochastic event buffeted by numerous environmental parameters (Xu et al. 2018). A slew of potential autochthonous starter cultures must be examined and the right fit identified to standardize and control the process, especially in large-scale cucumber fermentations (Jampaphaeng et al. 2018).

Generally, LAB constitute a small part (10^2^–10^3^ cfu/g) of the full spectrum of autochthonous microbiota of raw vegetables (Kothari et al. 2020). *Lactobacillus* spp., *Leuconostoc* spp., and *Pediococcus* spp. are the leading LAB predominating on the cucumber surface and are responsible for fermenting it (Behera et al. 2020). Finding plentiful *L. plantarum* is expected since it is the predominant *Lactobacillus* associated with SF cucumber because of its penchant for thriving in high acidity and salinity (Behera et al. 2018)*.*

A multitude of LAB spp. such as *L. brevis*, *S. thermophilus*, *E. faecium, P. pentosaceus,* and *Leuconostoc mesenteroides* in association with the fermented vegetable matter as seen here is in line with the notion where a broad vegetal microbial diversity could be attributed to a slew of agro-technological factors such as cultivars, geography, seasons, and aspects of the fermentation process like the quality of the base material used and the equipment surfaces (Khalil et al. 2018)*. Leuconostoc mesenteroides* is an infrequent primary fermenter of cucumber whose numbers are eventually superseded by *L. plantarum,* which finishes the process (Fusco et al. 2017). A greater abundance of *Enterobacteriaceae* was observed on conventionally farmed vegetables than organically farmed via culturing technique (Leff and Fierer 2013). This effect could be attributed to several factors: growing location, fertilizer use, pesticide use, other agricultural practices, and shipping and handling procedures (Beuchat et al. 2006). Enterococcal species as *E. hirae*, *E. faecium,* and *E. cloacae,* although naturally present on cucumber, are pinned down during the LAB-driven primary fermentation phase, only rebounding when the pH starts to rise. Since *E. cloacae* tends to spoil fermenting cucumbers, its presence is considered undesirable (Franco and Pérez-Díaz 2013).

Before assaying for probiotic functionality in candidate strains, their safety status was determined *in vitro* as recommended by FAO (Food et al. 2006) since probiotic starter cultures are consumed live in large dosages through the fermented product; therefore, their harmlessness to public health must be ensured (Chokesajjawatee et al. 2020). LAB spp. generally have a good safety record for human consumption; however, there are some exceptions involving *L. plantarum* (Cannon et al. 2005) and the enterococcal species *E. faecium* and *E. faecalis* (Sanchez Valenzuela et al. 2013; Strateva et al. 2016). LAB that are amino biogenic during spontaneous lactic acid fermentation can jeopardize the quality and safety of fermented foods (Alan et al. 2018), thus are ill-suited for use as starters and adjuncts (Behera et al. 2020; Belicová et al. 2013). Both candidate starters and probiotics must incontrovertibly demonstrate an absence of harmful enzymatic activities, such as β-glucosidase and β-glucuronidase, known to cause detrimental effects in the colon (Zielińska et al. 2015). Testing for virulence attributes such as hemolysins, typically associated with pathogens, was also deemed essential because of a past precedent of its occurrence, albeit a sporadic one in some *Lactobacillus* species (Domingos-Lopes et al. 2017), including *L. plantarum* strains of vegetable origin (Benítez-Cabello et al. 2019) and global regulatory guidelines emphatic stance on excluding probiotic candidates with even a smidgen of hemolytic behavior (Food et al. 2006). Enzyme profiling is also helpful for selecting strains to be used in food, as their enzymatic activities may affect the quality of the product (Colombo et al. 2018).

In recent years, it has been posited that food bacteria may act as reservoirs of antibiotic resistance genes, which might be transferred to gut commensals or pathogens (Hummel et al. 2007). However, *Lactobacillus,* especially those of fermented food origin, are unlikely to contribute to the AMR (Antimicrobial Resistance) problem in humans (Ma et al. 2017). Nevertheless, the European Food Safety Authority (EFSA) considers its assessment a primary criterion for according “qualified presumption of safety” (QPS) status (Zielińska et al. 2015). Intrinsic resistance to antibiotics such as aminoglycosides and vancomycin in *Pediococcus* (Shukla and Goyal 2014) and *Lactobacillus* species such as *L. plantarum* are intrinsic, non-transferable, and not sufficiently concerning to prevent their use for food fermentation (Abriouel et al. 2015; EFSA 2018). Therefore, all strains carrying antibiotic-resistant genes were adjudged as unsafe and precluded from further study. A high incidence of antibiotic resistance in *Lactobacillus* species has been attributed to insufficient checks and balances for the use and disposal of antibiotics in developing societies (Ma et al. 2017).

The probiotic effectiveness of these innocuous candidate starters was gauged by testing their tolerance to human gut physiological parameters such as pH, and bile tolerance must be examined in vitro (Anandharaj et al. 2015). Such an approach is faster, cheaper, reproducible, and offers more choice of controlled conditions disencumbered with many ethical restrictions associated with *in vivo* experimentation (Calvo-Lerma et al. 2019).

Robust acid tolerance in a potential probiotic starter candidate, besides empowering it to survive the human host’s GIT milieu, also prolongs its survivability in highly acidic fermented foods (Michalak et al. 2020). Tolerating extreme acidity as seen in *L. plantarum* strains NPL 1258, NPL 1259, and NPL 1280 is likely due to their adaptiveness to the pickle habitat (Adebayo-tayo and Onilude 2008) from where they have been isolated in this study. Withstanding bile exposure is vital for an ingested probiotic to survive in the small intestine (Anandharaj et al. 2015). Tolerating 0.15–0.3% concentration of bile salts is a sufficient threshold for any probiotic taken orally (Alp and Aslim 2010). The extent to which *Lactobacilli* can withstand bile is crucial since its levels in the gut are not static, fluctuating from 1.5 to 2% (w/v) in the first hour of digestion and eventually plateauing out at 0.3% (w/v) (Bao et al. 2010). LAB strains of vegetable origins are generally less bile resistant (Chiu et al. 2008), contrasting with our findings where some *L. plantarum* strains were significantly bile resistant. The adaptation mechanism to bile salts is multifactorial. It is attributed to changes in the bacterium’s ability to ferment carbohydrates, exopolysaccharides production, the balance of proteins and fatty acids in its cell membrane, and the ability to firmly adhere to human mucus (Ali et al. 2020).

Any prospective probiotics added to a food matrix should tolerate food processing and storage conditions and the entire swathe of gastrointestinal transit following ingestion right up to their intestinal site of action (Campos et al. 2019). A high titer of probiotic bacteria in food products at the point of consumption does not guarantee the same numbers in the gut because stomach acidity and intestinal bile can dramatically reduce their viability (da Cruz Rodrigues et al. 2019). Estimating the ravages of a GIT transit can be best done by simulating digestive processes *in vitro* (Campos et al. 2019), starting from the mouth to the ileum, and factoring in the effects of the food matrix, enzymes, and peristalsis (Neffe-Skocińska et al. 2018). Aside from its simplicity, low cost, and high throughput format, the clincher in this method is sequential exposure to acid and bile, a more authentic depiction of actual physiological events. Our findings of *L. plantarum* and *Pediococcus* strains capable of withstanding GIT transit without any significant deleteriousness support previous reports (Barbosa et al. 2015; Gheziel et al. 2019). *L. plantarum* derived from SF cucumber are likely to be intrinsically strong acid-tolerant because the pickled vegetables mimic harsh gastrointestinal conditions with pH values reaching three or lower (Cao et al. 2019).

One of the caveats of a good probiotic is that the strain must also endure the action of toxic metabolites (primarily phenols) produced during the digestion process (Jawan et al. 2019), which is also observed here. The selected *L. plantarum* and *P. pentosaceus* strains harbored a significant ability to metabolize food phenolics via active tannase and gallate decarboxylases, degrading tannin and gallic acids. Both are anti-nutritional factors (ANF) that inactivate digestive enzymes, insolubilize proteins, and affect the utilization of vitamins and minerals (Sáez et al. 2018). Microbial metabolization of nitrite during cucumber fermentation is also advantageous since dietary nitrite is linked to gastrointestinal cancers (Behera et al. 2020), and its control is important from a food safety standpoint (Ren et al. 2014).

LAB cells that strongly aggregate and whose surfaces are hydrophobic can adhere well to intestinal epithelia and occlude gut luminal pathogens (Abbasiliasi et al. 2017). BATH values are usually a proxy for autoaggregative strength (Lee et al. 2014). However, the extent to which cell hydrophobicity correlates with self and coaggregation can vary in LAB spp. (Li et al. 2015). Coaggregation is advantageous because it allows a probiotic strain to produce antimicrobial substances in proximity to several pathogen cells, effectively eliminating them from the GIT (Tuo et al. 2013).

Among the technological properties much sought in LAB are EPS production, salt tolerance, and antimicrobial metabolites. The ability of LAB to produce EPS is a common trait of LAB starters as it helps to improve the colonization of probiotic bacteria in the gastrointestinal tract and protect against the baleful effects of harmful resident bacteria (Kumar et al. 2017). Our finding of copiously EPS-producing *L. plantarum* strains from fermented cucumber has some precedent (Jiang et al. 2016).

The antimicrobial activity of LAB may improve the quality of fermented foods by eliminating spoilage and pathogenic bacteria (Michalak et al. 2020). Our results align with previous findings where *L. plantarum* strains inhibited gram-positive bacteria such as *S. aureus*, *S. pyogenes,* and *B. cereus,* and Gram-negative *C. freundii, E. coli,* and *P. aeruginosa* (Gheziel et al. 2019; Jiang et al. 2016). Indeed, antagonistic activity against *E. coli* is a relevant screening criterion because of the frequent presence of coliforms in cucumber pickle brine (Lu et al. 2013). Other food quality indicator microorganisms are also essential to test due to their high load on cucumber surfaces. *Pediococcus* species such as *P. pentosaceus* are inhibitory of pernicious and ubiquitous human pathogens such as *L. monocytogenes,* which has been challenging to control with standard industrial approaches (Huang et al. 2009).

Since cucumber fermentation occurs in brine with 5–7% NaCl (Di Cagno et al. 2008), candidate LAB starter strains ought to be halotolerant, as is the case here (Rodriguez-Palacios et al. 2017). Some strains of *L. plantarum* were exceptionally tolerant of salt, which may be due to the accumulation of osmo and cryoprotective solutes such as betaine and carnitine (Yao et al. 2020). Halotolerance is not a staple characteristic of the species as much variability is observed (Ziadi et al. 2019).

Carbohydrate utilization by lactic acid bacteria (LAB) defines the extent of cucumber fermentations, their quality, and long-term stability. Aside from glucose and fructose, alternate energy sources such as trehalose, cellobiose, and xylose, available in cucumber fermentations. These compounds remained present in cucumber fermentations even after glucose and fructose were consumed. The removal of these alternate energy sources by starter cultures during the most active period of the bioconversion prevents the proliferation of spoilage-associated microbes such as *L. buchneri* (Ucar et al. 2020a; Ucar et al. 2020b)*.* The strains of *L. plantarum* and *P. pentosaceus* used in this study can use trehalose and cellobiose and hamper the growth and metabolic activity of spoilage-associated microbes. Our *L. plantarum* and *P. pentosaceus* strains can utilize plant-based carbohydrates such as mannitol and inulin (Gustaw et al. 2018; Lee et al. 2014), which helps to promote the growth of *Lactobacillus*. The raffinose-degrading ability of our strains is an appealing feature because of their association with flatulence and GI disturbances in humans (Arunraj et al. 2020). Malonate utilization seems absent in all *L. plantarum* and *P. pentosaceus* strains associated with bloating during cucumber fermentation (Bintsis 2018). Our strains’ diverse metabolic profile helps them endure well in various non-dairy food matrices and the human intestine (Gupta and Bajaj 2018).

An extracellular enzyme produced by the candidate probiotics in the food matrix may improve the organoleptic properties of food and enhance human digestion (Gupta and Bajaj 2018). Enzymatic profiles of LAB that lack proteinases but have strong peptidase and esterase lipase activities are associated with imparting typical and desirable flavors to fermented vegetable products (Abbasiliasi et al. 2017; Goswami et al. 2017); therefore, the presence of these traits in our strains confirms their essential role in flavor development of fermented cucumber. Acid phosphatase and phosphohydrolase allow the probiotic strains to metabolize diverse substrates of the GIT (Shokryazdan et al. 2017). The absence of harmful enzymes, α-glucosidases or β-glucuronidase, implicated in carcinogenesis (Yeo et al. 2016), and chymotrypsin and N-acetyl-B-glucosaminidase that are linked with GIT dysfunction (Delgado et al. 2007) renders strains safer for human consumption.

Strains with copious EPS production with significant halotolerance and anti-pathogen capabilities were also assessed for withstanding oxidative stress. The antioxidative mechanisms protect colonizing LAB from attack by free radicals while benefiting the human host by mitigating cardiovascular diseases, diabetes, and ulcers of the GI tract (Ren et al. 2014). It was found that some strains of *L. plantarum* registered more antioxidant capacity than the reference strain. Vegetable matrices are typically characterized as high antioxidant environments (Verni et al. 2017). An aspect that has not gone unnoticed since fermented vegetables’ consumption was found to correlate with a lower COVID-19 pandemic mortality rate (Fonseca et al. 2020).

The proteolytic activity of LAB enhances the organoleptic profile of fermented foods through the release of free amino acids and their derivatives (Karasu et al. 2010; Verni et al. 2017). In addition, they are also involved in the degradation of allergenic and anti-nutritional proteins and the increased protein digestibility (Rizzello et al. 2016). A weakly proteolytic behavior of LAB species found in this study could be tied to their vegetal origins that are poor in protein content (Sáez et al. 2018).

Bile salt hydrolase (BSH) activity is essential for bacteria to thrive in the intestine and for the human host who can benefit from its cholesterol-lowering effect (Jones et al. 2013). BSH hydrolyzes conjugated glycodeoxycholic and taurodeoxycholic acid into glyco- and tauro-bile acids, respectively (Anandharaj et al. 2015), thereby protecting gut microbiota and probiotic bacteria from their toxicity (Shukla and Goyal 2014). Typically, LAB with vegetal origins where bile salts are absent do not possess bile salt hydrolase activity (Zielińska et al. 2015) which is at odds with our finding. Nevertheless, both *L. plantarum* and *P. pentosaceus* have been shown to have BSHs active against tauroconjugates of bile salts (Lee et al. 2014). This study supports previous contentions where LAB with active bile salt hydrolase has been shown to lower cholesterol levels, removing secondary bile salts and cholesterol from the human body (Peres et al. 2014). The extent to which they are successful is highly strain dependent (Zhang et al. 2014).

Inulin, fructooligosaccharides, and maltodextrins are well-established prebiotics that remain undigested in the upper gastrointestinal tract and are only fermented by *Lactobacillus* in the colon (Choudhary et al. 2019). Incorporating them into a probiotic carrying food matrix should have a proliferative effect on probiotic LAB, which is the case here. LAB species have cell-associated glycosidases and fructofuranosidases for hydrolysis of these substrates and using the resulting monomers as an energy source (Perrin et al. 2001). Choosing the right prebiotic is deemed essential and must be empirically determined for any novel synbiotic to succeed. We chose inulin instead of FOS as the prebiotic part of the synbiotic for lacto-fermentation of cucumber because the former was more readily fermentable and improved colonization and persistence of *L. plantarum* (Brajdes and Vizireanu 2013). The FOS, on the other hand, is more suitable as prebiotic to bifidobacterial instead of *Lactobacillus spp.* in synbiotic combinations (Schrezenmeir and de Vrese 2001).

When considering starters tailored for cucumber fermentation, the selection of strains from its natural microbiota allows for by-passing the adaptation challenges that allochthonous cultures could face, thus facilitating an improved nutritional, functional, and technological profile of fermented cucumber (Verni et al. 2017). *L. plantarum* and *Pediococcus* spp. are well-recognized starter cultures giving various fermented vegetable products (Behera et al. 2018). Although strains could be selected based on their technological features, multiple attributes are more helpful in making foods that have better sensory properties. A principal component analysis (PCA) can facilitate screening out the most suitable strain for fermenting vegetables (Sáez et al. 2018).

Mixed species inoculation of brined cucumbers with *P. pentosaceus* and *L. plantarum* has the potential advantage of an early, rapid initial growth and moderate acid production by the former species and a higher final acidity resulting from the lower pH tolerance of the latter (Tamang and Tamang 2010).

The typical industry standard of a minimum of 10^6^ cfu/g of probiotics in a product stems from clinical investigations where a minimum of 10^8^–10^9^ cfu/g of probiotic was deemed necessary for health benefits (da Cruz et al. 2009). Maintenance of appropriate numbers of probiotics during the storage of fermented vegetables is quite challenging due to the low pH of brine, nutrient depletion, and the accumulation of lactic acid (Valero-Cases and Frutos 2017). The supplementation of a natural plant origin prebiotic such as inulin can stimulate beneficial bacteria’s growth and metabolic activity in trying conditions (Nilchian et al. 2016). It can also protect the cells from refrigeration-associated cell damage, mainly through physical immobilization of the cells in inulin macroaggregates (Bedani et al. 2013). Before fermentation, the addition of prebiotics improves the shelf life viability of probiotic bacteria mixed in with a food product (Szydłowska and Kołożyn-Krajewska 2019). The pro-proliferative and protective effect of inulin in *L. plantarum* seems strain-dependent judging from reports in its favor (Valero-Cases and Frutos 2015) and disfavor (Nazzaro et al. 2012).

Starters bring about a rapid decrease of pH, which helps to reduce the risk of spoilage at the beginning of fermentation (Nilchian et al. 2016). The ability to acidify rapidly is desirable for any would-be LAB starter of vegetable fermentation (Wakil et al. 2014). The ability to acidify a medium is typical of many LAB species because of organic acids, mainly lactic acid (Greifova 2007). *L. plantarum* directed acidification of the samples has been applied for food preservation (Muthusamy et al. 2020). Optimizing brine concentration empirically as done here is critical for a desirable and appropriately fermented cucumber (Nilchian et al. 2016). Higher concentrations of brine promote spoilage because of lowered pH (Bautista-Gallego et al. 2010).

Lactic acid and acetic acid are the primary metabolic end products of carbohydrate fermentation during cucumber fermentation by LAB, which lowers the food pH, imbuing it with desirable organoleptic properties and eliminating pathogens, ensuring safety and stability of the final product (Adesulu-Dahunsi et al. 2018). A significant increase in lactic acid production during anaerobic fermentation of cucumber is associated with the capability of *L. plantarum* for making substantial lactic acid through its exclusive homofermentative pathway (Güney and Güngörmüşler 2020). The production of acetic acid was less than the lactic acid in all inoculated cucumber samples. The decreased concentrations of lactic acid accompanied by increased acetic acid concentrations in the control fermented cucumber sample could be due to the degradation of lactic acid into acetic acid by spoilage-associated microorganisms, especially *L. buchneri* (Johanningsmeier and McFeeters 2013).

Sensory analysis is an indispensable tool to determine consumer acceptability (Cuffia et al. 2018). Irrespective of a product’s health claims, the customer will likely reject it if the sensory profile is poor (Karimi et al. 2012). We used an affective sensory method using a 5-point hedonic scale popular in the industry and academic research (Greifova 2007). The concept is serially monadic that does not allow for retasting or contextual reference to capture consumer purchase behavior more accurately (Wichchukit and O'Mahony 2015). Sensory analysis is a human-centric propriocentric view of a food product. Taste and pleasure are among the most meaningful predictors of food choice (Brunsø et al. 2002). Lacto-fermented cucumbers with mixed starter cultures were preferable to monoculture fermented cucumber concerning taste and pleasure. This difference might be because mixed strain cultures are relatively less affected by vicissitudes of handling, storage, and applications and contribute more to desirable flavors while cutting down on unpleasant ones (Holzapfel 2002). Bitterness and over sourness are negatively hedonistic (Greifova 2007), and mar acceptance of control spontaneously fermented cucumbers (Drewnowski and Gomez-Carneros 2000; Verheul et al. 2013).

## Conclusion

The present study highlights the capacity of two LAB strains, autochthonous to SF cucumber, as potential probiotic cum starter culture candidates. The results indicate that inoculated selected starters manifested *in vitro* several desirable, beneficial probiotic attributes such as antioxidant, BSH activity, cholesterol assimilation, and antibiotic susceptibility. In addition, the inoculated starters remained significantly viable during fermentation and contributed to the aroma and flavor of the fermented cucumber. They prevented putrescence caused by spoilage bacteria and enhanced the sensorial aspects of the fermented product. A high inoculum of *L. plantarum* and *P. pentosaceus* strains (NPL 1258 and NPL 1259) was found to control the fermented cucumber’s quality effectively. Strain probioticity, no doubt a good and valuable attribute, nonetheless does not obviate the need to examine the fermentation kinetics and physiological benefits further before they could be recommended for large-scale commercial application.

## References

[CR1] Abbasiliasi S, Tan JS, Bashokouh F, Ibrahim TAT, Mustafa S, Vakhshiteh F, Sivasamboo S, Ariff AB (2017). In vitro assessment of *Pediococcus acidilactici* Kp10 for its potential use in the food industry. BMC Microbiol.

[CR2] Abriouel H, Muñoz MCC, Lerma LL, Montoro BP, Bockelmann W, Pichner R, Kabisch J, Cho G-S, Franz CM, Galvez A (2015). New insights in antibiotic resistance of *Lactobacillus* species from fermented foods. Food Res Int.

[CR3] Adebayo-tayo BC, Onilude AA (2008). Screening of lactic acid bacteria strains isolated from some Nigerian fermented foods for EPS production. World Appl Sci J.

[CR4] Adesulu-Dahunsi AT, Jeyaram K, Sanni AI, Banwo K (2018). Production of exopolysaccharide by strains of *Lactobacillus plantarum* YO175 and OF101 isolated from traditional fermented cereal beverage. PeerJ.

[CR5] Alan Y, Topalcengiz Z, Dığrak M (2018). Biogenic amine and fermentation metabolite production assessments of *Lactobacillus plantarum* isolates for naturally fermented pickles. LWT-Food Sci Technol.

[CR6] Ali SA, Singh P, Tomar SK, Mohanty AK, Behare P (2020). Proteomics fingerprints of systemic mechanisms of adaptation to bile in *Lactobacillus fermentum*. J Proteome.

[CR7] Alp G, Aslim B (2010). Relationship between the resistance to bile salts and low pH with exopolysaccharide (EPS) production of *Bifidobacterium* spp. isolated from infants feces and breast milk. Anaerobe.

[CR8] Anandharaj M, Sivasankari B, Santhanakaruppu R, Manimaran M, Rani RP, Sivakumar S (2015). Determining the probiotic potential of cholesterol-reducing *Lactobacillus* and *Weissella* strains isolated from gherkins (fermented cucumber) and south Indian fermented koozh. Res Microbiol.

[CR9] Arunraj R, Skori L, Kumar A, Hickerson NM, Shoma N, Samuel MA (2020). Spatial regulation of alpha-galactosidase activity and its influence on raffinose family oligosaccharides during seed maturation and germination in Cicer arietinum. Plant Signal Behav.

[CR10] Bao Y, Zhang Y, Zhang Y, Liu Y, Wang S, Dong X, Wang Y, Zhang H (2010). Screening of potential probiotic properties of *Lactobacillus fermentum* isolated from traditional dairy products. Food Control.

[CR11] Barbosa J, Borges S, Teixeira P (2015). *Pediococcus acidilactici* as a potential probiotic to be used in food industry. Int J Food Sci Technol.

[CR12] Bautista-Gallego J, Arroyo-López FN, Durán-Quintana M, Garrido-Fernández A (2010). Fermentation profiles of Manzanilla-Aloreña cracked green table olives in different chloride salt mixtures. Food Microbiol.

[CR13] Bedani R, Rossi EA, Saad SMI (2013). Impact of inulin and okara on *Lactobacillus acidophilus* La-5 and *Bifidobacterium animalis* Bb-12 viability in a fermented soy product and probiotic survival under *in vitro* simulated gastrointestinal conditions. Food Microbiol.

[CR14] Behera SS, El Sheikha AF, Hammami R, Kumar A (2020). Traditionally fermented pickles: how the microbial diversity associated with their nutritional and health benefits?. J Funct Foods.

[CR15] Behera SS, Ray RC, Zdolec N (2018). *Lactobacillus plantarum* with functional properties: an approach to increase safety and shelf-life of fermented foods. Biomed Res Int.

[CR16] Belicová A, Mikulášová M, Dušinský R (2013) Probiotic potential and safety properties of *Lactobacillus plantarum* from Slovak Bryndza cheese. Biomed Res Intdoi:10.1155/2013/760298, 2013, 1, 8 10.1155/2013/760298 PMC3777194 24093103

[CR17] Benítez-Cabello A, Calero-Delgado B, Rodríguez-Gómez F, Garrido-Fernández A, Jiménez-Díaz R, Arroyo-López FN (2019). Biodiversity and multifunctional features of lactic acid bacteria isolated from table olive biofilms. Front Microbiol.

[CR18] Beuchat L, Rvon Holy A, Lindsay D (2006). Vectors and conditions for preharvest contamination of fruits and vegetables with pathogens capable of causing enteric diseases. Br Food J.

[CR19] Bintsis T (2018). Lactic acid bacteria as starter cultures: an update in their metabolism and genetics. AIMS Microbiol.

[CR20] Brajdes C, Vizireanu C (2013). Stability of *Lactobacillus plantarum* from functional beverage–based sprouted backwheat in the conditions simulating in the upper gastrointestinal tract. Glob Res Anal.

[CR21] Brunsø K, Grunert KG, Fjord TA (2002) Consumers’ food choice and quality perception vol 77. MAPP, Center for markedsovervågning,-vurdering og-bearbejdning til Aarhus School of Business, MAPP - Centre for Research on Customer Relations in the Food Sector, 2002

[CR22] Calvo-Lerma J, Fornés-Ferrer V, Heredia A, Andrés A (2019). *In vitro* digestion models to assess lipolysis: the impact of the simulated conditions of gastric and intestinal pH, bile salts and digestive fluids. Food Res Int.

[CR23] Campos PA, Martins EMF, Martins ML, de Oliveira Martins AD, Júnior BRdCL, da Silva RR, Trevizano LM (2019). In vitro resistance of *Lactobacillus plantarum* LP299v or *Lactobacillus rhamnosus* GG carried by vegetable appetizer. LWT.

[CR24] Cannon J, Lee T, Bolanos J, Danziger L (2005). Pathogenic relevance of *Lactobacillus*: a retrospective review of over 200 cases. Eur J Clin Microbiol Infect Dis.

[CR25] Cao Z, Pan H, Li S, Shi C, Wang S, Wang F, Ye P, Jia J, Ge C, Lin Q (2019). *In vitro* evaluation of probiotic potential of lactic acid bacteria isolated from Yunnan De’ang pickled tea. Probiotics Antimicrob Proteins.

[CR26] Chiu HH, Tsai CC, Hsih HY, Tsen HY (2008). Screening from pickled vegetables the potential probiotic strains of lactic acid bacteria able to inhibit the *Salmonella* invasion in mice. J Appl Microbiol.

[CR27] Chokesajjawatee N, Santiyanont P, Chantarasakha K, Kocharin K, Thammarongtham C, Lertampaiporn S, Vorapreeda T, Srisuk T, Wongsurawat T, Jenjaroenpun P (2020). Safety assessment of a nham starter culture *Lactobacillus plantarum* BCC9546 via whole-genome analysis. Sci Rep.

[CR28] Choudhary S, Singh M, Sharma D, Attri S, Sharma K, Goel G (2019). Principal component analysis of stimulatory effect of synbiotic combination of indigenous probiotic and inulin on antioxidant activity of soymilk. Probiotics Antimicrob Proteins.

[CR29] Colombo M, Castilho NP, Todorov SD, Nero LA (2018). Beneficial properties of lactic acid bacteria naturally present in dairy production. BMC Microbiol.

[CR30] Cuffia F, Bergamini C, Candioti M (2018). Probiotic soft sheep’s cheese: evaluation of probiotic survival and its influence on proteolysis and organoleptic characteristics. Int Food Res J.

[CR31] da Cruz AG, Buriti FCA, de Souza CHB, Faria JAF, Saad SMI (2009). Probiotic cheese: health benefits, technological and stability aspects. Trends Food Sci Technol.

[CR32] da Cruz Rodrigues VC, da Silva LGS, Simabuco FM, Venema K, Antunes AEC (2019). Survival, metabolic status and cellular morphology of probiotics in dairy products and dietary supplement after simulated digestion. J Funct Foods.

[CR33] Dash SK, Chakraborty SP, Mandal D, Roy S (2012). Isolation and characterization of multi drug resistant uropathogenic *Escherichia coli* from urine sample of urinary tract infected patients. Int J Life Sci Pharma Res.

[CR34] Dec M, Urban-Chmiel R, Stępień-Pyśniak D, Wernicki A (2017). Assessment of antibiotic susceptibility in *Lactobacillus* isolates from chickens. Gut Pathog.

[CR35] Delgado S, O'sullivan E, Fitzgerald G, Mayo B (2007). Subtractive screening for probiotic properties of *Lactobacillus* species from the human gastrointestinal tract in the search for new probiotics. J Food Sci.

[CR36] Di Cagno R, Coda R, De Angelis M, Gobbetti M (2013). Exploitation of vegetables and fruits through lactic acid fermentation. Food Microbiol.

[CR37] Di Cagno R, Surico RF, Siragusa S, De Angelis M, Paradiso A, Minervini F, De Gara L, Gobbetti M (2008). Selection and use of autochthonous mixed starter for lactic acid fermentation of carrots, French beans or marrows. Int J Food Microbiol.

[CR38] Domingos-Lopes M, Stanton C, Ross P, Dapkevicius M, Silva C (2017). Genetic diversity, safety and technological characterization of lactic acid bacteria isolated from artisanal Pico cheese. Food Microbiol.

[CR39] Drewnowski A, Gomez-Carneros C (2000). Bitter taste, phytonutrients, and the consumer: a review. Am J Clin Nutr.

[CR40] EFSA (2018). Guidance on the characterisation of microorganisms used as feed additives or as production organisms. EFSA J.

[CR41] Fonseca S, Rivas I, Romaguera D, Quijal M, Czarlewski W, Vidal A, Fonseca J, Ballester J, Anto J, Basagana X (2020) Association between consumption of fermented vegetables and COVID-19 mortality at a country level in Europe. MedRxiv. 10.1101/2020.07.06.20147025

[CR42] Food, Organization A, Food, Nations AOotU, Organization WH, FAO/WHO. J (2006). Probiotics in food: health and nutritional properties and guidelines for evaluation.

[CR43] Franco W, Pérez-Díaz I (2013). Microbial interactions associated with secondary cucumber fermentation. J Appl Microbiol.

[CR44] Fusco V, Oguntoyinbo FA, Franz CM (2017) Fermentation to improve food security in Africa and Asia. In: Alexandru Mihai Grumezescu AMH (ed) Soft Chemistry and Food Fermentation. Academic Press, pp 337–378. 10.1016/B978-0-12-811412-4.00012-6

[CR45] Gardner NJ, Savard T, Obermeier P, Caldwell G, Champagne CP (2001). Selection and characterization of mixed starter cultures for lactic acid fermentation of carrot, cabbage, beet and onion vegetable mixtures. Int J Food Microbiol.

[CR46] Garmasheva I, Vasyliuk O, Kovalenko N, Oleschenko L (2019). New approach for fast screening of lactic acid bacteria for vegetable fermentation. J Microbiol Biotechnol Food Sci.

[CR47] Gheziel C, Russo P, Arena MP, Spano G, Ouzari H-I, Kheroua O, Saidi D, Fiocco D, Kaddouri H, Capozzi V (2019). Evaluating the probiotic potential of *Lactobacillus plantarum* strains from algerian infant feces: towards the design of probiotic starter cultures tailored for developing countries. Probiotics Antimicrob Proteins.

[CR48] Goswami G, Bora SS, Parveen A, Boro RC, Barooah M (2017). Identification and functional properties of dominant lactic acid bacteria isolated from Kahudi, a traditional rapeseed fermented food product of Assam, India. J Ethn Foods.

[CR49] Greifova ZKJKM (2007). Analytical and organoleptic profiles of lactic acid fermented cucumber juice with addition of onion juice. J Food Nutr Res.

[CR50] Guan Q, Xiong T, Xie M (2020). Influence of probiotic fermented fruit and vegetables on human health and the related industrial development trend. Engineering.

[CR51] Güney D, Güngörmüşler M (2020). Development and comparative evaluation of a novel fermented juice mixture with probiotic strains of lactic acid bacteria and *Bifidobacteria*. Probiotics Antimicrob Proteins.

[CR52] Guo H, Pan L, Li L, Lu J, Kwok L, Menghe B, Zhang H, Zhang W (2017). Characterization of antibiotic resistance genes from *Lactobacillus* isolated from traditional dairy products. J Food Sci.

[CR53] Gupta M, Bajaj BK (2018). Functional characterization of potential probiotic lactic acid bacteria isolated from kalarei and development of probiotic fermented oat flour. Probiotics Antimicrob Proteins.

[CR54] Gustaw K, Michalak M, Polak-Berecka M, Waśko A (2018). Isolation and characterization of a new fructophilic *Lactobacillus plantarum* FPL strain from honeydew. Ann Microbiol.

[CR55] Halder D, Mandal M, Chatterjee SS, Pal NK, Mandal S (2017). Indigenous probiotic *Lactobacillus* isolates presenting antibiotic like activity against human pathogenic bacteria. Biomedicines.

[CR56] Holzapfel W (2002). Appropriate starter culture technologies for small-scale fermentation in developing countries. Int J Food Microbiol.

[CR57] Huang Y, Luo Y, Zhai Z, Zhang H, Yang C, Tian H, Li Z, Feng J, Liu H, Hao Y (2009). Characterization and application of an anti-Listeria bacteriocin produced by *Pediococcus pentosaceus* 05-10 isolated from Sichuan Pickle, a traditionally fermented vegetable product from China. Food Control.

[CR58] Huang Y, Wang X, Wang J, Wu F, Sui Y, Yang L, Wang Z (2013). *Lactobacillus plantarum* strains as potential probiotic cultures with cholesterol-lowering activity. J Dairy Sci.

[CR59] Hummel AS, Hertel C, Holzapfel WH, Franz CM (2007). Antibiotic resistances of starter and probiotic strains of lactic acid bacteria. Appl Environ Microbiol.

[CR60] Jampaphaeng K, Ferrocino I, Giordano M, Rantsiou K, Maneerat S, Cocolin L (2018). Microbiota dynamics and volatilome profile during stink bean fermentation (Sataw-Dong) with *Lactobacillus plantarum* KJ03 as a starter culture. Food Microbiol.

[CR61] Jawan R, Kasimin M, Jalal S, Faik AM, Abbasiliasi S, Ariff A (2019) Isolation, characterisation and in vitro evaluation of bacteriocin-producing lactic acid bacteria from fermented products of Northern Borneo for their beneficial roles in food industry. In: Journal of Physics: Conference Series, Kota Kinabalu, Sabah, Malaysia, 2-3 October 2018, vol 1. IOP Publishing, p 012020. 10.1088/1742-6596/1358/1/012020

[CR62] Ji Y, Kim H, Park H, Lee J, Lee H, Shin H, Kim B, Franz CM, Holzapfel WH (2013). Functionality and safety of lactic bacterial strains from Korean kimchi. Food Control.

[CR63] Jiang M, Zhang F, Wan C, Xiong Y, Shah NP, Wei H, Tao X (2016). Evaluation of probiotic properties of *Lactobacillus plantarum* WLPL04 isolated from human breast milk. J Dairy Sci.

[CR64] Johanningsmeier SD, McFeeters RF (2013). Metabolism of lactic acid in fermented cucumbers by *Lactobacillus buchneri* and related species, potential spoilage organisms in reduced salt fermentations. Food Microbiol.

[CR65] Jones ML, Tomaro-Duchesneau C, Martoni CJ, Prakash S (2013). Cholesterol lowering with bile salt hydrolase-active probiotic bacteria, mechanism of action, clinical evidence, and future direction for heart health applications. Expert Opin Biol Ther.

[CR66] Kaktcham PM, Temgoua J-B, Zambou FN, Diaz-Ruiz G, Wacher C, de Lourdes P-CM (2018). *In vitro* evaluation of the probiotic and safety properties of bacteriocinogenic and non-bacteriocinogenic lactic acid bacteria from the intestines of *Nile Tilapia* and common carp for their use as probiotics in aquaculture. Probiotics Antimicrob Proteins.

[CR67] Karasu N, Şimşek Ö, Çon AH (2010). Technological and probiotic characteristics of *Lactobacillus plantarum* strains isolated from traditionally produced fermented vegetables. Ann Microbiol.

[CR68] Karimi R, Sohrabvandi S, Mortazavian A (2012). Sensory characteristics of probiotic cheese. Compr Rev Food Sci Food Saf.

[CR69] Khalil ES, Manap A, Yazid M, Mustafa S, Alhelli AM, Shokryazdan P (2018). Probiotic properties of exopolysaccharide-producing *Lactobacillus* strains isolated from Tempoyak. Molecules.

[CR70] Kothari D, Lee W-D, Jung ES, Niu K-M, Lee CH, Kim S-K (2020). Controlled fermentation using autochthonous *Lactobacillus plantarum* improves antimicrobial potential of Chinese chives against poultry pathogens. Antibiotics.

[CR71] Kumar V, Kumari A, Angmo K, Bhalla TC (2017). Isolation and characterization of lactic acid bacteria from traditional pickles of Himachal Pradesh, India. J Food Sci Technol.

[CR72] Kumari A, Angmo K, Bhalla TC (2016). Probiotic attributes of indigenous *Lactobacillus* spp. isolated from traditional fermented foods and beverages of north-western Himalayas using in vitro screening and principal component analysis. J Food Sci Technol.

[CR73] Lee KW, Park JY, Sa HD, Jeong JH, Jin DE, Heo HJ, Kim JH (2014). Probiotic properties of *Pediococcus* strains isolated from jeotgals, salted and fermented Korean sea-food. Anaerobe.

[CR74] Leff JW, Fierer N (2013). Bacterial communities associated with the surfaces of fresh fruits and vegetables. PLoS One.

[CR75] Li Q, Liu X, Dong M, Zhou J, Wang Y (2015). Aggregation and adhesion abilities of 18 lactic acid bacteria strains isolated from traditional fermented food. Int J Agric Policy Res.

[CR76] Lu HJ, Breidt F, Pérez-Díaz I (2013). Development of an effective treatment for a 5-log reduction of *Escherichia coli* in refrigerated pickle products. J Food Sci.

[CR77] Ma Q, Fu Y, Sun H, Huang Y, Li L, Yu Q, Dinnyes A, Sun Q (2017). Antimicrobial resistance of *Lactobacillus* spp. from fermented foods and human gut. LWT.

[CR78] Michalak M, Kubik-Komar A, Waśko A, Polak-Berecka M (2020). Starter culture for curly kale juice fermentation selected using principal component analysis. Food Biosci.

[CR79] Montaño A, Casado FJ, De Castro A, Sánchez AH, Rejano L (2004). Vitamin content and amino acid composition of pickled garlic processed with and without fermentation. J Agric Food Chem.

[CR80] Mukherjee PK, Nema NK, Maity N, Sarkar BK (2013). Phytochemical and therapeutic potential of cucumber. Fitoterapia.

[CR81] Muthusamy K, Soundharrajan I, Srisesharam S, Kim D, Kuppusamy P, Lee KD, Choi KC (2020). Probiotic characteristics and antifungal activity of *Lactobacillus plantarum* and its impact on fermentation of Italian ryegrass at low moisture. ApplSci.

[CR82] Nazzaro F, Fratianni F, Orlando P, Coppola R (2012). Biochemical traits, survival and biological properties of the probiotic *Lactobacillus plantarum* grown in the presence of prebiotic inulin and pectin as energy source. Pharmaceuticals.

[CR83] Neffe-Skocińska K, Rzepkowska A, Szydłowska A, Kołożyn-Krajewska D (2018) Trends and possibilities of the use of probiotics in food production. In: Holban AM, Grumezescu AM (eds) Alternative and replacement foods. Academic Press, pp 65-94. doi:10.1016/B978-0-12-811446-9.00003-4

[CR84] Nel S, Davis SB, Endo A, Dicks LM (2020). Phylogenetic analysis of *Leuconostoc* and *Lactobacillus* species isolated from sugarcane processing streams. MicrobiologyOpen.

[CR85] Nilchian Z, Sharifan A, Rahimi E, Mazid Abadi N (2016). Improvement of fermented cucumber characteristics by starter culture of *Lactobacillus plantarum*, *L. bulgaricus* and *S. thermophiles*. J Food Biosi Technol.

[CR86] Peres CM, Alves M, Hernandez-Mendoza A, Moreira L, Silva S, Bronze MR, Vilas-Boas L, Peres C, Malcata FX (2014). Novel isolates of *lactobacilli* from fermented Portuguese olive as potential probiotics. LWT-Food Sci Technol.

[CR87] Perrin S, Warchol M, Grill J, Schneider F (2001). Fermentations of fructo-oligosaccharides and their components by *Bifidobacterium infantis* ATCC 15697 on batch culture in semi-synthetic medium. J Appl Microbiol.

[CR88] Reina LD, Breidt F, Fleming HP, Kathariou S (2005). Isolation and selection of lactic acid bacteria as biocontrol agents for nonacidified, refrigerated pickles. J Food Sci.

[CR89] Ren D, Li C, Qin Y, Yin R, Du S, Ye F, Liu C, Liu H, Wang M, Li Y (2014). *In vitro* evaluation of the probiotic and functional potential of *Lactobacillus* strains isolated from fermented food and human intestine. Anaerobe.

[CR90] Rizzello CG, Tagliazucchi D, Babini E, Rutella GS, Saa DLT, Gianotti A (2016). Bioactive peptides from vegetable food matrices: research trends and novel biotechnologies for synthesis and recovery. J Funct Foods.

[CR91] Rodriguez-Palacios A, Staempfli HR, Weese JS (2017) High doses of halotolerant gut-indigenous *Lactobacillus plantarum* reduce cultivable Lactobacilli in newborn calves without increasing its species abundance. Int J Microbiol. doi:10.1155/2017/2439025, 2017, 1, 11 10.1155/2017/2439025 PMC5449734 28596790

[CR92] Sáez GD, Flomenbaum L, Zárate G (2018). Lactic acid bacteria from argentinean fermented foods: isolation and characterization for their potential use as starters for fermentation of vegetables. Food Technol Biotechnol.

[CR93] Sanchez Valenzuela A, Lavilla Lerma L, Benomar N, Gálvez A, Perez Pulido R, Abriouel H (2013). Phenotypic and molecular antibiotic resistance profile of *Enterococcus faecalis* and *Enterococcus faecium* isolated from different traditional fermented foods. Foodborne Pathog Dis.

[CR94] Schrezenmeir J, de Vrese M (2001). Probiotics, prebiotics, and synbiotics—approaching a definition. Am J Clin Nutr.

[CR95] Shokryazdan P, Jahromi M, Liang J, Sieo C, Kalavathy R, Idrus Z, Ho Y (2017). In vitro assessment of bioactivities of *Lactobacillus* strains as potential probiotics for humans and chickens. J Food Sci.

[CR96] Shukla R, Goyal A (2014). Probiotic potential of *Pediococcus pentosaceus* CRAG3: a new isolate from fermented cucumber. Probiotics Antimicrob Proteins.

[CR97] Sim K, Cox MJ, Wopereis H, Martin R, Knol J, Li M-S, Cookson WO, Moffatt MF, Kroll JS (2012). Improved detection of *Bifidobacteria* with optimised 16S rRNA-gene based pyrosequencing. PLoS One.

[CR98] Songisepp E, Hütt P, Rätsep M, Shkut E, Kõljalg S, Truusalu K, Stsepetova J, Smidt I, Kolk H, Zagura M (2012). Safety of a probiotic cheese containing *Lactobacillus plantarum* Tensia according to a variety of health indices in different age groups. J Dairy Sci.

[CR99] Strateva T, Atanasova D, Savov E, Petrova G, Mitov I (2016). Incidence of virulence determinants in clinical *Enterococcus faecalis* and *Enterococcus faecium* isolates collected in Bulgaria. Braz J Infect Dis.

[CR100] Szydłowska A, Kołożyn-Krajewska D (2019). Development of potentially probiotic and synbiotic pumpkin frozen desserts. CYTA J Food.

[CR101] Taheri H, Moravej H, Tabandeh F, Zaghari M, Shivazad M (2009). Screening of lactic acid bacteria toward their selection as a source of chicken probiotic. Poult Sci.

[CR102] Tamang B, Tamang JP (2010). In situ fermentation dynamics during production of gundruk and khalpi, ethnic fermented vegetable products of the Himalayas. Indian J Microbiol.

[CR103] Tomaro-Duchesneau C, Jones ML, Shah D, Jain P, Saha S, Prakash S (2014). Cholesterol assimilation by *Lactobacillus* probiotic bacteria: an *in vitro* investigation. Biomed Res Int.

[CR104] Tuo Y, Yu H, Ai L, Wu Z, Guo B, Chen W (2013). Aggregation and adhesion properties of 22 *Lactobacillus* strains. J Dairy Sci.

[CR105] Ucar RA, Pérez-Díaz IM, Dean LL (2020). Content of xylose, trehalose and l-citrulline in cucumber fermentations and utilization of such compounds by certain lactic acid bacteria. Food Microbiol.

[CR106] Ucar RA, Pérez-Díaz IM, Dean LL (2020). Gentiobiose and cellobiose content in fresh and fermenting cucumbers and utilization of such disaccharides by lactic acid bacteria in fermented cucumber juice medium. Food Sci Nutr.

[CR107] Valero-Cases E, Frutos MJ (2015). Effect of different types of encapsulation on the survival of *Lactobacillus plantarum* during storage with inulin and *in vitro* digestion. LWT-Food Sci Technol.

[CR108] Valero-Cases E, Frutos MJ (2017). Effect of inulin on the viability of *L. plantarum* during storage and *in vitro* digestion and on composition parameters of vegetable fermented juices. Plant Foods Hum Nutr.

[CR109] Verheul M, Slimestad R, Johnsen L (2013). Physicochemical changes and sensory evaluation of slicing cucumbers from different origins. Europ J Hort Sci.

[CR110] Verni M, Wang C, Montemurro M, De Angelis M, Katina K, Rizzello CG, Coda R (2017). Exploring the microbiota of faba bean: functional characterization of lactic acid bacteria. Front Microbiol.

[CR111] Wakil S, Laba S, Fasika S (2014). Isolation and identification of antimicrobial-producing lactic acid bacteria from fermented cucumber. Afr J Biotechnol.

[CR112] Weiss G, Jespersen L (2010). Transcriptional analysis of genes associated with stress and adhesion in *Lactobacillus acidophilus* NCFM during the passage through an *in vitro* gastrointestinal tract model. J Mol Microbiol Biotechnol.

[CR113] Wichchukit S, O'Mahony M (2015). The 9-point hedonic scale and hedonic ranking in food science: some reappraisals and alternatives. J Sci Food Agric.

[CR114] Xu X, Luo D, Bao Y, Liao X, Wu J (2018). Characterization of diversity and probiotic efficiency of the autochthonous lactic acid bacteria in the fermentation of selected raw fruit and vegetable juices. Front Microbiol.

[CR115] Yao W, Yang L, Shao Z, Xie L, Chen L (2020). Identification of salt tolerance-related genes of *Lactobacillus plantarum* D31 and T9 strains by genomic analysis. Ann Microbiol.

[CR116] Yeo S, Lee S, Park H, Shin H, Holzapfel W, Huh CS (2016). Development of putative probiotics as feed additives: validation in a porcine-specific gastrointestinal tract model. Appl Microbiol Biotechnol.

[CR117] Zago M, Fornasari ME, Carminati D, Burns P, Suàrez V, Vinderola G, Reinheimer J, Giraffa G (2011). Characterization and probiotic potential of *Lactobacillus plantarum* strains isolated from cheeses. Food Microbiol.

[CR118] Zhai Y, Pérez-Díaz IM, Diaz JT (2018). Viability of commercial cucumber fermentation without nitrogen or air purging. Trends Food Sci Technol.

[CR119] Zhang J, Zhang X, Zhang L, Zhao Y, Niu C, Yang Z, Li S (2014). Potential probiotic characterization of *Lactobacillus plantarum* strains isolated from Inner Mongolia “Hurood” cheese. J Microbiol Biotechnol.

[CR120] Ziadi M, Bouzaiene T, Lakhal S, Zaafouri K, Massoudi S, Dousset X, Hamdi M (2019). Screening of lactic starter from Tunisian fermented vegetables and application for the improvement of caper (*Capparis spinosa*) fermentation through an experimental factorial design. Ann Microbiol.

[CR121] Zielińska D, Rzepkowska A, Radawska A, Zieliński K (2015). *In vitro* screening of selected probiotic properties of *Lactobacillus* strains isolated from traditional fermented cabbage and cucumber. Curr Microbiol.

[CR122] Zieliński H, Surma M, Zielińska D (2017) The naturally fermented sour pickled cucumbers. In: Frías J, Martínez-Villaluenga C, Peñas E (eds) Fermented Foods in Health and Disease Prevention. Elsevier, pp 503–516. 10.1016/B978-0-12-802309-9.00021-2

